# Pyrrolidinium and Imidazolium-Based Ionic Liquids as Electrolytes for Na_0.67_Ni_0.33_Mn_0.67_O_2_ Positive Electrode for Sodium–Ion Batteries

**DOI:** 10.1021/acs.jpcb.5c07871

**Published:** 2026-01-28

**Authors:** Leandro S. Domingues, Roberto M. Torresi, Vincent Vivier, Mireille Turmine, Vitor L. Martins, Hercilio G. de Melo

**Affiliations:** † Departamento de Engenharia Metalúrgica e de Materiais, 28133 Universidade de São Paulo, Av. Professor Mello Moraes, 2463—Butantã, São Paulo, São Paulo 05508-030, Brazil; ‡ Laboratoire de Réactivité de Surface (LRS), 27063 Sorbonne Université, CNRS, 4 Place Jussieu, Paris 75005, France; § Departamento de Química Fundamental, Instituto de Química, Universidade de São Paulo, Av. Prof. Lineu Prestes 748—Butantã, São Paulo, São Paulo 05508-000, Brazil

## Abstract

In this paper, the electrochemical performance of two nitrogen-based ionic liquids (ILs), 1-butyl-1-methylpyrrolidinium bis­(trifluoromethylsulfonyl)­imide (BMPyr-TFSI) and 2,3-dimethylimidazolium bis­(trifluoromethylsulfonyl)­imide (BMMI-TFSI), with different concentrations of NaTFSI, as electrolytes for the Na_0.67_Ni_0.33_Mn_0.67_O_2_ (NNM) positive electrode for sodium–ion batteries (SIBs) were compared with the conventional 1.0 mol L^–1^ NaClO_4_ in carbonate electrolyte. Moreover, the influence of salt concentration on the physicochemical properties of both ILs was evaluated. Amidst the neat ILs, BMPyr-TFSI showed better transport properties than BMMI-TFSI, whereas, for NaTFSI-mixtures, adding salt was detrimental to the ILs’ properties. The poorer transport properties of the ILs compared to those of the carbonate electrolyte negatively impact the NNM electrode performance. At C/10, the highest discharge capacity obtained in IL mixtures was 40 mA h g^–1^ for BMPyr-TFSI with 0.5 mol L^–1^ of NaTFSI, compared to 59 mA h g^–1^ for NNM in NaClO_4_ electrolyte. Lowering the current density improved the performance of NNM in both BMPyr and BMMI-based mixtures, achieving specific capacities and Coulombic efficiencies above 53 mA h g^–1^ and 96%, respectively, at C/50. This approach has proven effective in overcoming the kinetic limitations due to the poorer transport properties displayed by ILs, encouraging the implementation of these electrolytes in SIBs.

## Graphical abstract

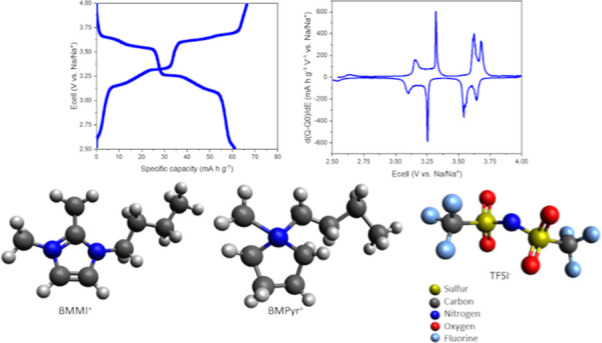

## Introduction

The environmental impact of producing energy from the combustion of fossil fuels has encouraged research into renewable energy sources and, at the same time, increased the demand for appropriate energy storage systems.^1^ Secondary batteries have emerged as an interesting prospect, with lithium-ion batteries (LIBs) being the most prominent. However, the concentration of Li reserves in a few countries and their ongoing extraction have reduced its availability, which could lead to increased costs and supply shortages.^2–4^ Moreover, current extraction methods pose some environmental risks.^5^ As a result, replacing Li with other materials that have similar properties is becoming a critical issue. One promising approach is the use of sodium–ion batteries (SIBs), leveraging sodium’s abundance and its chemical similarity to lithium.^3,6^

Over the years, various materials have been studied for use as positive electrodes in SIBs, in which layered oxide systems are the most widespread due to their structural flexibility and versatility.^7,8^ Their intrinsic structure allows better diffusion of sodium ions, thereby enhancing rate capability.^7–9^ One potential layered oxide material commonly studied for use as the positive electrode in SIBs is Na_0.67_Ni_0.33_Mn_0.67_O_2_ (NNM). Depending on the oxygen stacking ordering, this compound can exhibit two different structures: prismatic (P) and octahedral (O)-type.^7^ Although octahedral-type cathodes typically exhibit a higher Na storage capacity compared to prismatic systems, they are reported to suffer from poor rate capability and inferior cycling stability.^10^ Conversely, the crystalline arrangement of the prismatic structure enables faster Na^+^ diffusion due to the presence of open pathways.^11^ Moreover, the P2-type compound (2 stands for the number of repeated oxygen layers within the unit cell) has a specific theoretical capacity of 173 mA h g^–1^ (assuming the transfer of 2/3 mol of Na^+^), which is comparable to some layered oxides used in lithium-ion systems.^12,13^ However, studies on this material in the literature primarily focus on changes in electrode composition, rather than on suitable electrolytes.^14–19^ These latter play a critical role in the battery performance since they facilitate ionic conduction between the electrodes in a cell.

In addition to high ionic conductivity, the electrolyte must display good thermal, chemical, and electrochemical stability, low reactivity and low corrosivity toward other cell components, and be environmentally friendly.^20,21^ In metal-ion systems, organic electrolytes are typically used as solvents. Nonetheless, due to fire hazard associated with their volatility,^22^ research into new electrolytes with properties similar to those of conventional systems is becoming important for SIBs. In this context, electrolytes based on ionic liquids (ILs) are excellent candidates. Their negligible flammability practically eliminates the risk of fire in batteries,^23^ promoting adequate safety levels for SIB operation. Moreover, this family of electrolytes can achieve conductivity comparable to conventional systems, along with exceptional chemical and electrochemical stability.^24–26^

Risthaus et al. published in 2018 one of the few articles on the implementation of ILs as substitutes for conventional organic systems for NNM.^27^ The authors evaluated the performance of the positive electrode in the presence of 1-butyl-1-methylpyrrolidinium bis­(trifluoromethylsulfonyl)­imide containing 1.0 mol L^–1^ NaTFSI, using galvanostatic charge/discharge cycles at 60 °C, scanning electron microscopy (SEM), X-ray diffraction (XRD), and X-ray absorption spectroscopy (sXAS). At 60 °C, the electrochemical results demonstrated enhanced electrode performance in the nitrogen-based electrolyte, achieving a higher specific capacity than experiments performed at room temperature using the conventional 1.0 mol L^–1^ NaPF_6_ in ethylene carbonate (EC)/dimethyl carbonate (DMC) (EC/DMC 1:1 v/v) electrolyte.

Despite the promising results obtained by Risthaus et al.,^27^ it is acknowledged that the performance of ILs in energy storage devices is also significantly influenced by the transport properties of the electrolyte.^28^ The selection of the optimum cation–anion combination,^29^ in conjunction with the concentration of salt in the electrolyte,^3,30^ play a fundamental role, as they affect ionic transport and energy supply rate. Thus, for better comprehension of the applicability of this family of electrolytes for NNM electrode material, it is essential to evaluate how these properties influence the battery’s performance.

The present work aims to evaluate the feasibility of two nitrogen-based ILs families, namely, 1-butyl-1-methylpyrrolidinium bis­(trifluoromethylsulfonyl)­imide (BMPyr-TFSI) and 1-butyl-2,3-dimethylimidazolium bis­(trifluoromethylsulfonyl)­imide (BMMI-TFSI), and their mixtures with different NaTFSI salt concentrations as electrolytes for NNM. The motivation for evaluating these two electrolyte families lies in their distinct properties. Imidazolium salts exhibit high chemical and electrochemical stability owing to the configuration of the central ring.^31,32^ In contrast, pyrrolidinium shows good transport properties attributable to its quasi-flat geometry, which hinders steric effects that are detrimental to ionic mobility.^33,34^ The investigation was performed using electrochemical techniques, specifically electrochemical impedance spectroscopy (EIS), to access information related to the electrode properties, and charging–discharging experiments at various C-rates to evaluate NNM performance in the different electrolytes. In order to provide a comprehensive assessment of the electrochemical tests, the physicochemical properties, density, viscosity, and conductivity for the different ILs were determined. In addition, the diffusion coefficient of Na^+^ in these ILs was estimated. As an additional analysis, the thermal properties of the selected ILs were analyzed.

## Methods

### Ionic Liquids

1-butyl-1-methylpyrrolidinium bis­(trifluoromethylsulfonyl)­imide (BMPyr-TFSI) and 1-butyl-2,3-dimethylimidazolium bis­(trifluoromethylsulfonyl)­imide (BMMI-TFSI) were selected as the nitrogen-based ILs. The former was commercially provided by Iolitec (99.9% purity), while the latter was synthesized according to previous reports.^25,35^ Sodium bis­(trifluoromethylsulfonyl)­imide (NaTFSI), Sigma-Aldrich (99.5% purity), was selected as the salt.

The different mixtures were prepared in an argon-filled glovebox (oxygen and water contents below 1.0 ppm) at varying salt concentrations: 0.5 and 1.0 mol L^–1^ for BMPyr-TFSI, and 0.5 and 0.83 mol L^–1^ for BMMI-TFSI. The maximum concentration of 0.83 mol L^–1^ for BMMI-TFSI corresponds to the solubility of this salt. Prior to the assembly of the batteries, the ILs were dried at 100 °C for 24 h on a hot plate within the glovebox. The water content of the electrolytes was measured using a Karl Fischer coulometric titrator (Metrohm) to ensure a maximum water content of less than 10 ppm.

### Physicochemical Properties

The physicochemical properties of the ILs were determined over a temperature range from 25 to 80 °C. Density and viscosity measurements were performed using a digital densimeter/viscometer (SVM 3000/G2, Anton Paar) with a thermoregulator. Ionic conductivity was measured by EIS, using an Autolab potentiostat (PGSTAT204, Metrohm) equipped with a Microcell HC 70 cell stand with a temperature controller (Peltier effect). The EIS spectra were obtained over a frequency range from 100 kHz to 10 mHz, with a perturbation amplitude of 10 mV and with six points acquired per decade. The cell constant was obtained using an aqueous solution of 0.01 mol L^–1^ KCl.

### Thermal Analysis

In order to access information on possible phase transitions on the different ILs and their mixtures with NaTFSI, differential scanning calorimetry (DSC) analyses were carried out using a T.A. Instruments—DSC Q10, coupled with a refrigerated cooling system (RCS) under nitrogen atmosphere. It is important to highlight that the neat IL BMMI-TFSI was not included in the analysis because its glass transition temperature (*T*_g_) is reported to be lower than the minimum temperature reached by the equipment (−80 °C).^25,36^ Prior to analysis, the samples for DSC were weighed and hermetically sealed within an aluminum pan inside the glovebox to avoid air exposure. The measurements were performed by initially cooling the samples (at a rate of 20 °C min^–1^) to −80 °C and maintaining them at this temperature for 2 min, and then heating at a rate of 20 °C min^–1^ up to 300 °C.

### Active Material Synthesis

NNM was selected as the positive electrode of the battery. The active material was synthesized by solid-state chemistry using the following precursors: anhydrous sodium carbonate (Na_2_CO_3_), Syth (99.5% purity); nickel­(II) acetate tetrahydrate (C_4_H_6_NiO_4_·4H_2_O), and manganese­(II) acetate tetrahydrate (Mn­(H_3_CCOO)_2_·4H_2_O), Sigma-Aldrich (98% and 99% purity, respectively). The precursors were weighed separately to ensure a stoichiometric ratio of 0.8:0.6:0.4 between Na, Mn, and Ni. Sodium excess was added to compensate for losses during sintering.^37^ The compounds were then mixed using an agate mortar and pestle, prior to being transferred into a muffle furnace (EGD3P-S) using a ceramic crucible. The sintering process was carried out in two stages. Initially, the powder was heated at 100 °C for 12 h to remove water, followed by a heat treatment up to 850 °C (at a rate of 2 °C min^–1^) for 12 h, and then cooled to room temperature in air. The sintering temperature was selected based on the results reported by Liu et al.^37^ A low heating rate was used to avoid the formation of NiO impurities in the active material.^18^ The final crystal structure was characterized by XRD using a Shimadzu XRD7000 diffractometer equipped with a Cu Kα source generated at 40 kV and 30 mA. The XRD spectrum of NNM powder was performed in the 2θ range from 10° to 80° and is presented in the Supporting Information section (Figure S1).

### Electrode Preparation and Battery Assembly

The positive electrode was prepared by mixing 90 wt % active material, 5 wt % polyvinylidene fluoride (PVDF) binder, and 5 wt % Super C_65_ conductive additive (500 mg of total solids). The slurry was prepared using the following procedure: the PVDF binder was dissolved in 1.5 mL of *N*-methyl-pyrrolidone (NMP) using an Eppendorf with eight ZrO_2_ spheres of 1.5 mm; then, both Super C_65_ and NNM powder were added to the Eppendorf, and the assembly was mixed using a ball mill (Solab) for 3 h at a frequency of 617 bpm. The final slurry was then doctor-bladed onto an aluminum foil (resulting in a final wet film thickness of 200 μm) and dried at 80 °C under vacuum for 24 h. The electrodes were punched into discs with a diameter of 16 mm with an average load mass of 5.13 mg cm^–2^. Prior to battery assembly, the electrode surface was characterized by SEM (FEG Inspect 50 scanning electron microscope). The SEM micrograph of the electrode and the energy dispersive spectroscopy (EDS) analysis are presented in the Supporting Information section (Figures S2 and S3, respectively).

CR2032 coin cells were assembled in an argon-filled glovebox, where the levels of water and oxygen were maintained below 1.0 ppm. The positive electrode consisted of NMM, while a 10 mm-diameter sodium disk served as the negative electrode. A 19 mm glass fiber (Whatman GF/F) was used as separator.

### Electrochemical Characterization

Electrochemical measurements were carried out using the BioLogic BCS-805 battery cycler. To evaluate electrode performance under different conditions, the batteries were galvanostatically charged and discharged over a potential range of 2.5–4.0 V vs Na/Na^+^ at varying C-rates (for the system under study, 1C is equivalent to 173 mA h g^–1^). The higher cell potential limit was chosen on the basis that NNM undergoes an irreversible phase transition when 2/3 mol of Na^+^ is removed from the crystal lattice, as previously reported.^38,39^ As an additional tool, the potential profiles obtained were analyzed using the differential capacity curves (d*Q*/d*E* vs *E*) to determine the effect of the different media in the intercalation/deintercalation process of Na^+^. The conventional electrolyte NaClO_4_ (1.0 mol L^–1^) in a 1:1 (v/v) mixture of EC and propylene carbonate (PC) was used for comparison. All the experiments were performed at room temperature (25 °C).

### Sodium Diffusion Coefficient Determination

EIS measurements were conducted to estimate the sodium diffusion coefficient in the different IL-based mixtures and in the conventional perchlorate electrolyte. All experiments were performed at room temperature, using a Swagelok cell with a 10 mm sodium disk as the counter and reference electrodes, and an 8 mm copper disk serving as the working electrode. The approach adopted in this study was analogous to that used by previous researchers^40–43^ prior to the acquisition of the EIS spectrum. The cells were initially subjected to a galvanostatic cathodic current density of 40 μA cm^–2^ to reach a stationary potential between sodium stripping from the counter electrode and its plating onto the copper electrode. After 1 h under these conditions, the system was left at the open circuit potential for an additional 1 h prior to the impedance measurement. The EIS spectra were obtained over a frequency range from 100 kHz to 10 mHz, with a perturbation amplitude of 10 mV and with six points acquired per frequency decade. The impedance data were fitted to determine the diffusion coefficient from the low-frequency region. The latter will be further discussed in the results and discussions section.

### CEI Properties

To provide further insights into the cycling results across different electrolytes, EIS experiments were conducted to assess the properties of the electrode and the cathode solid electrolyte interphase (CEI). For this purpose, fresh batteries (half-cell system) were assembled and subjected to galvanostatic charge and discharge cycles over the potential range of 2.5–4.0 V vs Na/Na^+^ with impedance spectra acquired before and after the cycles. For the conventional electrolyte, two cycles were carried out at C/20 (specific current of 8.65 mA g^–1^), while for the ILs mixtures, two cycles at C/50 (3.46 mA g^–1^) were carried out. All EIS diagrams were obtained using the same parameters described above.

## Results and Discussion

### Physicochemical Properties

Density is a critical parameter in the design of lighter energy storage systems,^44^ and its value is used to calculate the IL molar conductivity (Λ_m_).^24^ Figure 1 shows the density variation with temperature for the different ILs and their mixtures, showing that it decreases linearly with increasing temperature. Among the various electrolytes, the imidazolium-based cation shows a higher density than the pyrrolidinium counterpart. As demonstrated in previous studies,^25,45^ BMMI^+^ is less geometrically packed than BMPyr^+^ owing to the presence of a second methyl group on the imidazolium ring. This results in a larger volume (266 Å^3^ against 253 Å^3^). However, a more plausible explanation for the observed behavior is provided by the differences in molar mass between the two species: the molecular weight of BMMI^+^ is 153.25 g mol^–1^, while that of BMPyr^+^ is 142.27 g mol^–1^.

**Figure 1:**
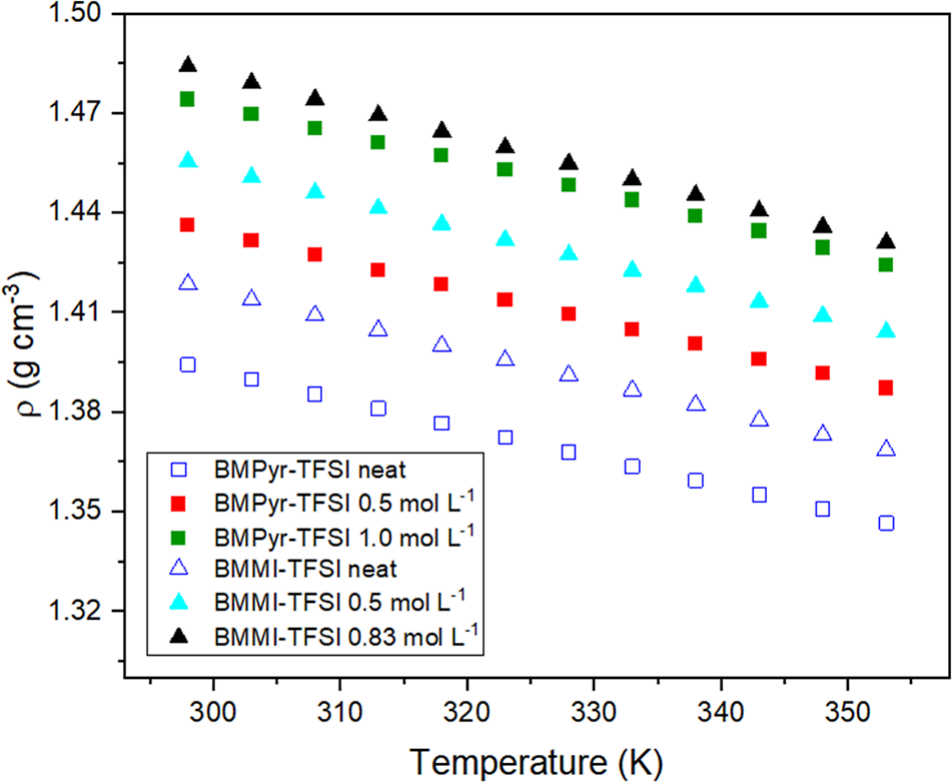
Density variation as a function of temperature for the different mixtures of nitrogen-based ILs.

The addition of the sodium salt has been demonstrated to increase the IL density for all electrolytes. As shown in Table 1, at 25 °C, the average increase for BMPyr-TFSI is 3.0% and 5.8% for 0.5 and 1.0 mol L^–1^ NaTFSI. In the case of BMMI-TFSI, the average increase is 2.6% and 5.1% for 0.5 and 0.83 mol L^–1^ NaTFSI, respectively. This outcome was anticipated, as the addition of alkali metal salts to ILs results in ion clustering, accompanied by changes in ion packing, thus contributing to an increase in overall density.^46,47^

**Table 1:** Density, Viscosity, and Conductivity of the Nitrogen-Based ILs at 25 °C.

cation–anion pair	NaTFSI concentration (mol L^–1^)	density (g cm^–3^)	viscosity (mPa s)	conductivity (mS cm^–1^)
BMPyr-TFSI	--	1.394	79.3	2.64
	0.5	1.436	173.6	1.31
	1.0	1.474	357.5	0.58
BMMI-TFSI	--	1.418	99.9	1.96
	0.5	1.455	189.2	1.15
	0.83	1.491	301.8	0.83

Figure 2 displays the Arrhenius plots of the viscosity (η) (Figure 2a) and conductivity (σ) (Figure 2b) for the pure ILs and their mixtures with NaTFSI, which were fitted using the Vogel–Fulcher–Tammann (VFT) eqs (eqs 1 and 2). VFT is based on the Arrhenius equation; however, it is regarded as a more suitable method for liquids undergoing a glass transition transformation;^48^ it is, therefore, commonly used to analyze viscosity and conductivity data for ILs.^25,49–52^ Moreover, it generally provides a better fit than the Arrhenius and Litovitz equations.^53–56^

$$\eta =\eta_{0}\;e\left(\frac{D_{VFT}\times T_{0}}{T-{\;T}_{0}}\right)$$ 1

$$\sigma =\sigma_{0}\;e\left(\frac{-D_{VFT}\times T_{0}}{T-{\;T}_{0}}\right)$$ 2

In eq 1 and 2, η_0_ and σ_0_ are the pre-exponential factors, *T* and *T*_0_ are the absolute temperature and the “ideal glass transition temperature”, respectively.^57^ *D*_VFT_ corresponds to the fragility of the IL, and refers to changes in the property produced by temperature.^50,58^ The high values obtained indicate that the properties show reduced susceptibility to temperature fluctuations, signifying a less fragile liquid state.^49^ The parameters from the fitting procedure are presented in the Supporting Information section (Tables S1 and S2).

**Figure 2:**
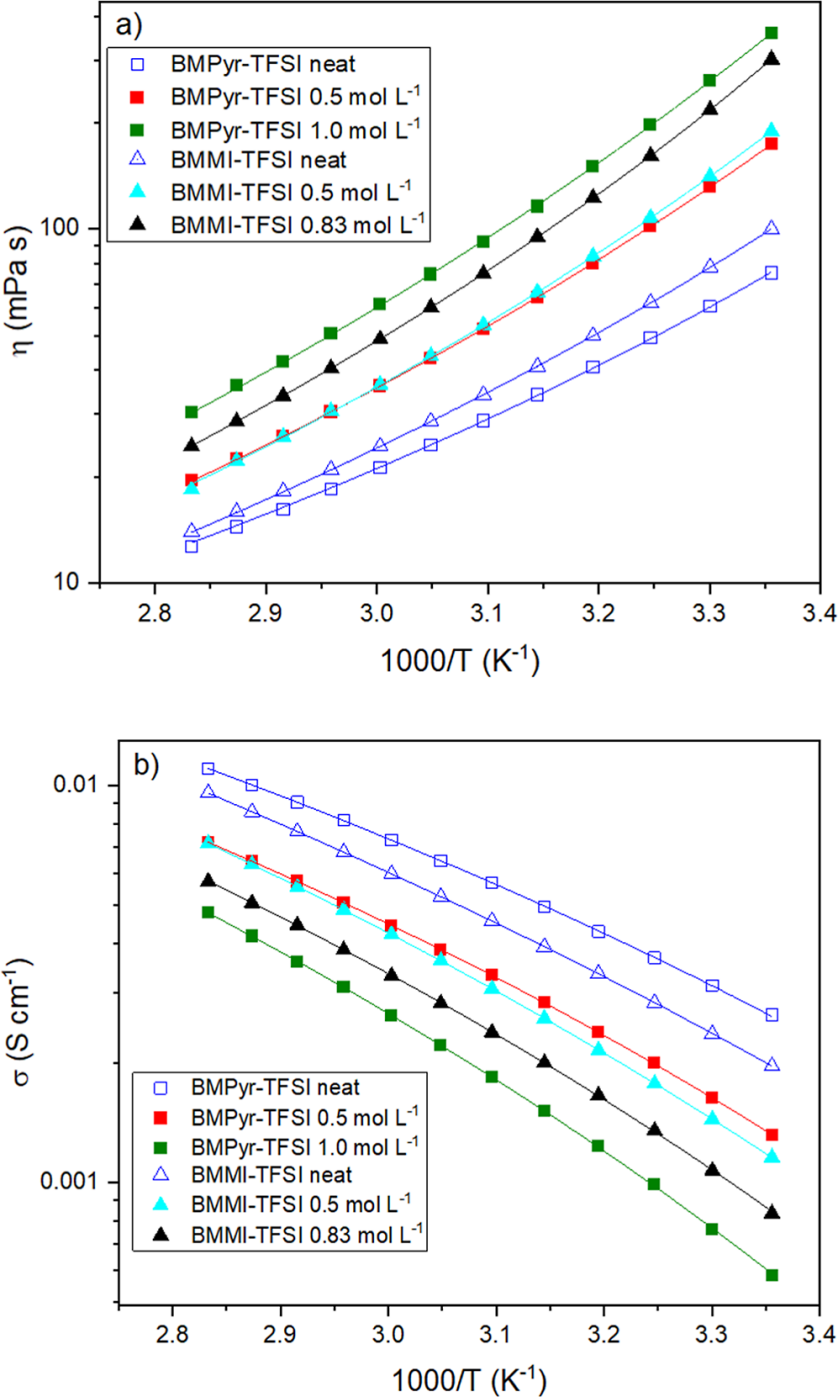
Arrhenius plots for the variation of viscosity and conductivity as a function of temperature for the different mixtures of nitrogen-based ILs (the symbols stand for the experimental data and the lines for the fitting with the VFT equations): (a) viscosity; (b) conductivity.

Viscosity and conductivity play major roles in the design of electrolytes for batteries, showing a reciprocal relationship: that is, an increase in viscosity corresponds to a decrease in conductivity. The former is associated with the liquid's flow resistance, while the latter includes the mobility of charge carriers. These two parameters can be influenced by the intrinsic characteristics of the IL cation and anion, such as their nature, size, structure, chemical functionalities, molecular weight, chain flexibility, and the presence of heteroatoms.^24,29,45,49,59–63^ Accordingly, the selection of the cation and anion combination is a crucial factor, as it can either diminish or augment these interactions and, consequently, affect the transport properties of the IL.^63^

Concerning the neat ILs, BMMI-TFSI (blue triangle) showed a higher viscosity (Figure 2a) and a lower conductivity (Figure 2b) than the pyrrolidinium-based cation, BMPyr-TFSI (blue square). As previously reported by other authors, the inherent planarity of the BMMI^+^ ring favors ion–ion interactions,^25^ thereby increasing the flow resistance. Moreover, the best transport properties displayed by BMPyr-TFSI can be attributed to its quasi-flat geometry,^33^ which mitigates steric effects that could impede ionic mobility.^34^ An examination of the mixture of salts reveals a correlation between the increase in NaTFSI concentration and the concomitant increase in η and decrease in σ. As shown in Table 1, for BMPyr^+^ electrolytes, at 25 °C, the viscosity increases 2.19 and 4.51 times when 0.5 and 1.0 mol L^–1^ of NaTFSI were added to the electrolytes. At the same time, conductivity decreases by 0.50 and 0.78 times from the initial value. For BMMI^+^ electrolytes, the increase in η was 1.9 and 3.0 times with the addition of 0.5 and 0.83 mol L^–1^ of NaTFSI, whereas σ shows a decrease of 0.41 and 0.58 times the value obtained for the neat IL. This same trend was confirmed in other studies exploring the physicochemical properties of IL-based electrolytes with different salt concentrations.^3,30,36,50,64–68^ For NaTFSI-based systems, the sodium ion tends to coordinate with three TFSI^–^ anion, resulting in the formation of [Na­(TFSI)_3_]^2–^, as demonstrated by Monti et al.^30^ using Raman spectroscopy and DFT calculations. Since the charge density of Na^+^ is higher compared to organic cations, Na-TFSI interactions are enhanced as the number of ionic species increases in the medium.^69–71^ The formation of more [Na­(TFSI)_3_]^2–^ aggregates impairs the transport properties.

An interesting methodology for examining the physicochemical characteristics of different IL families and collecting data on their ionicity is to correlate the molar conductivity, Λ_m_, with the fluidity (η^–1^), which is referred to as Walden’s plot. The ionicity of an IL is particularly significant because it is directly associated with the availability of charge carriers.^72^ To increase the ionicity, it is imperative that both the cation and the anion exhibit minimal coordination capacity. This, in turn, leads to an increase in the density of species that are responsible for the transport properties. The formation of ionic pairs can result in the creation of neutral aggregates, reducing the ionicity of the IL.^73^

Figure 3 shows a log–log representation of Walden’s plot, while its linear representation is shown in the Supporting Information section (Figure S4). For a very dilute solution (dotted reference line in Figure 3), an ideal behavior is expected, corresponding to total ionization. All the tested liquids fall below this line, suggesting the existence of ionic associations resulting from aggregate formation.^73^ Interestingly, when comparing the two neat nitrogen families, BMPyr-TFSI is closer to the ideal dotted line in Figure 3 than BMMI-TFSI. In their study, Ramirez et al.^45^ used EIS to compare the ionic conductivity of BMMI-B­(CN_4_) and BMPyr-B­(CN_4_), focusing exclusively on the contribution of ionic species. They further used nuclear magnetic resonance (NMR) to estimate the contribution of all species to the overall conductivity. Through the calculation of the ratio between the two conductivities, it was ascertained that BMPyr has a higher ratio than BMMI, indicating that the former would have more species contributing to conductivity, thus suggesting that the imidazolium cation has a greater tendency to form neutral aggregates than BMPyr^+^, which is in agreement with our results. In the presence of the Na^+^ salt, the curves for BMPyr-based and BMMI-based electrolytes shifted, moving further away from the reference curve, a tendency that is accentuated with increasing salt concentration. This was an expected outcome, as increasing the concentrations of Na^+^ and TFSI^–^ in the medium must result in a notable increase in the frequency of interactions and the formation of aggregates, leading to a reduction in the electrolyte's ionicity.

**Figure 3:**
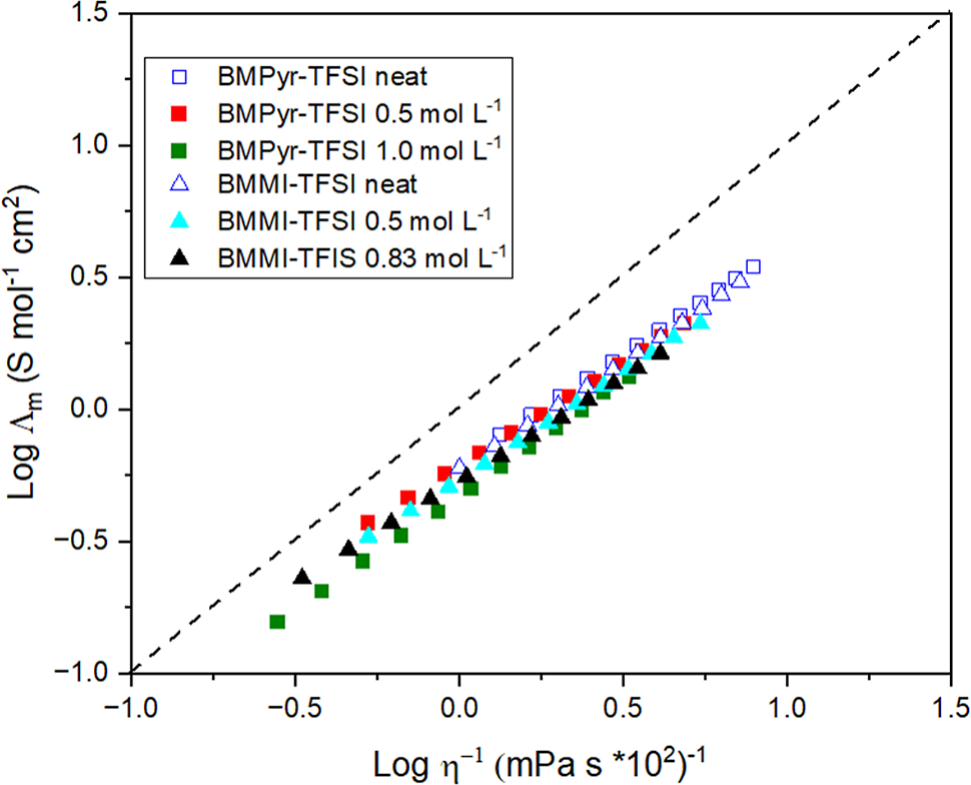
Log–log representation of Walden’s plot for the different mixtures of nitrogen-based ILs.

### Thermal Analysis

Figure 4 shows the results of the DSC investigation for the ILs and their mixtures with NaTFSI. Concerning the pyrrolidinium-based electrolytes, the DSC pattern for the neat IL (Figure 4a) reveals the existence of one exothermic peak, near −45 °C, and two endothermic peaks near −25 and −15 °C, which are in agreement with previous studies.^74,75^ The exothermic peak is associated with a crystalline phase,^74,75^ while the two endothermic peaks reflect the two stages of the melting of the IL.^75,76^ Given the reported *T*_g_ of BMPyr-TFSI, which is estimated to range from −87 ^75^ and −89 ^3^ °C, it is not expected to be observed in the thermogram. Consequently, the results are not included in the study.

**Figure 4:**
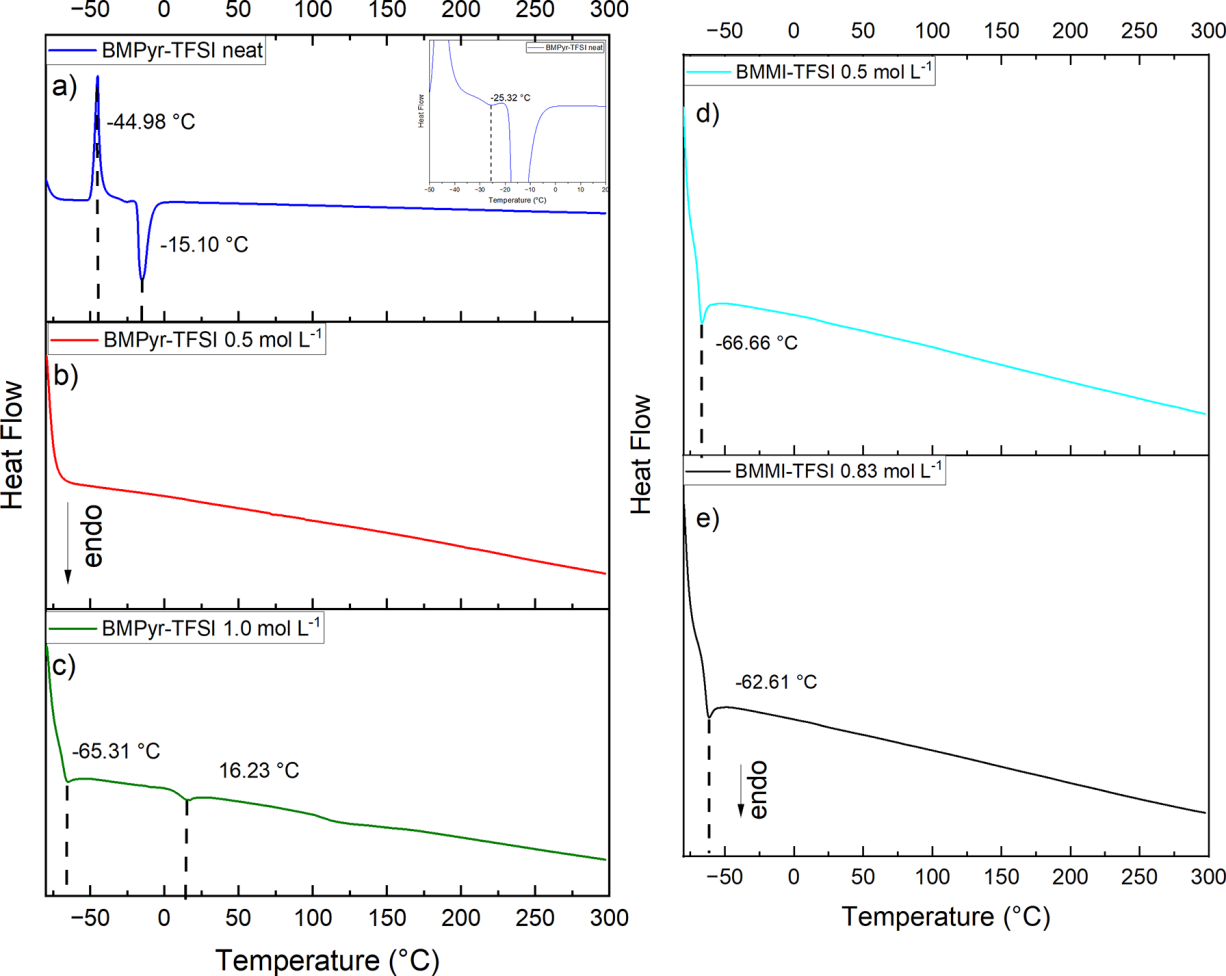
DSC patterns for the different ILs: (a) BMPyr-TFSI neat; (b) BMPyr-TFSI 0.5 mol L^–1^; (c) BMPyr-TFSI 1.0 mol L^–1^; (d) BMMI-TFSI 0.5 mol L^–1^; (e) BMMI-TFSI 0.83 mol L^–1^.

Adding NaTFSI changed the DSC profiles compared to the salt-free condition. For the lowest salt concentration (Figure 4b—red curve), no apparent peaks are observed, whereas for 1.0 mol L^–1^ (Figure 4c—green curve), two endothermic peaks, near −65 and 16 °C, are present. These changes can be ascribed to the disorder promoted by Na^+^ in the pyrrolidinium-based IL.^3,77,78^ As shown by Noor et al.,^3^ from 0.3 mol L^–1^ of NaTFSI the IL’s crystallization seems to be completely inhibited, and consequently, no endothermic peak associated with BMPyr-TFSI crystallization is expected to be observed. Moreover, the *T*_g_ tends to shift to higher temperatures as the salt concentration increases in the electrolyte, which can be associated with the formation of Na^+^ complexes and clusters.^77^ For instance, the *T*_g_ of a 0.5 mol L^–1^ of NaTFSI, is reported to be approximately −80 °C^3^, which accounts for the absence of its respective endothermic peak in Figure 4b. Conversely, for BMPyr-TFSI with 1.0 mol L^–1^ of NaTFSI (Figure 4c), the first endothermic peak could be correlated to the electrolyte’s *T*_g_, while the second one could be associated with the distinct conformers of TFSI^–^.^77^

For the BMMI-based mixtures (Figure 4d,e), a single endothermic peak is observed at each concentration. For 0.5 mol L^–1^ of NaTFSI (Figure 4d), the peak is situated at approximately −67 °C, while for 0.83 mol L^–1^ (Figure 4e), it is close to −63 °C. Both peaks can be associated with the electrolyte’s glass transition,^30,36^ similarly to what was previously reported for BMMI-TFSI mixtures containing LiTFSI salt.^36^ Similarly to the response for the BMPyr-based electrolyte, *T*_g_ increases with the Na^+^ concentration, indicating an increase in Na-ion interactions. When comparing the two families of ILs for the same salt concentration (0.5 mol L^–1^ of NaTFSI), a higher *T*_g_ temperature is observed for the imidazolium ion-based medium. This response suggests that the interactions generated by the sodium’s presence have a more significant impact on the dynamics of this electrolyte, consistent with its physicochemical properties.

### Electrochemical Characterization

Figure 5 shows the potential profiles for NNM (with a theoretical specific capacity of 173 mA h g^–1^) at different C-rates for the various electrolytes. For better visualization, only Figure 5e shows the corresponding specific currents for each C-rate. The potential curves in the conventional electrolyte (Figure 5a) exhibit four plateaus. During the discharge, these plateaus are located at about 3.10, 3.25, 3.50, and 3.60 V vs Na/Na^+^, whereas during charging, they are located at about 3.15, 3.30, 3.60, and 3.70 V vs Na/Na^+^. These plateaus agree with those observed in other NNM studies^18,37,79,80^ and are associated with redox reactions governed by Ni^2+^/Ni^3+^/Ni^4+^ couples, as verified by X-ray photoelectron spectroscopy analyses.^39,81,82^ XRD analyses performed by Lu et al.,^83^ Wen et al.,^84^ and Wang et al.^85^ show that NNM does not undergo phase transition in the potential range between 2.0 and 4.0 V vs Na/Na^+^ and remains in a prismatic structure. However, below 2.0 V vs Na/Na^+^, a redox reaction occurs between the Mn^3+^/Mn^4+^ redox couple, which can lead to lattice distortions in the electrode due to Jahn–Teller effect of Mn^3+^.^86,87^ The onset of a small potential plateau can be observed at about 2.5 V vs Na/Na^+^ during the discharge and at around 2.6 V vs Na/Na^+^ during charge. This plateau is associated with rearrangements between Na^+^ and the host vacancies.^82^ For currents above 34.5 mA g^–1^ (C/5), the absence of some plateaus is ascribed to increased polarization of the system.

**Figure 5:**
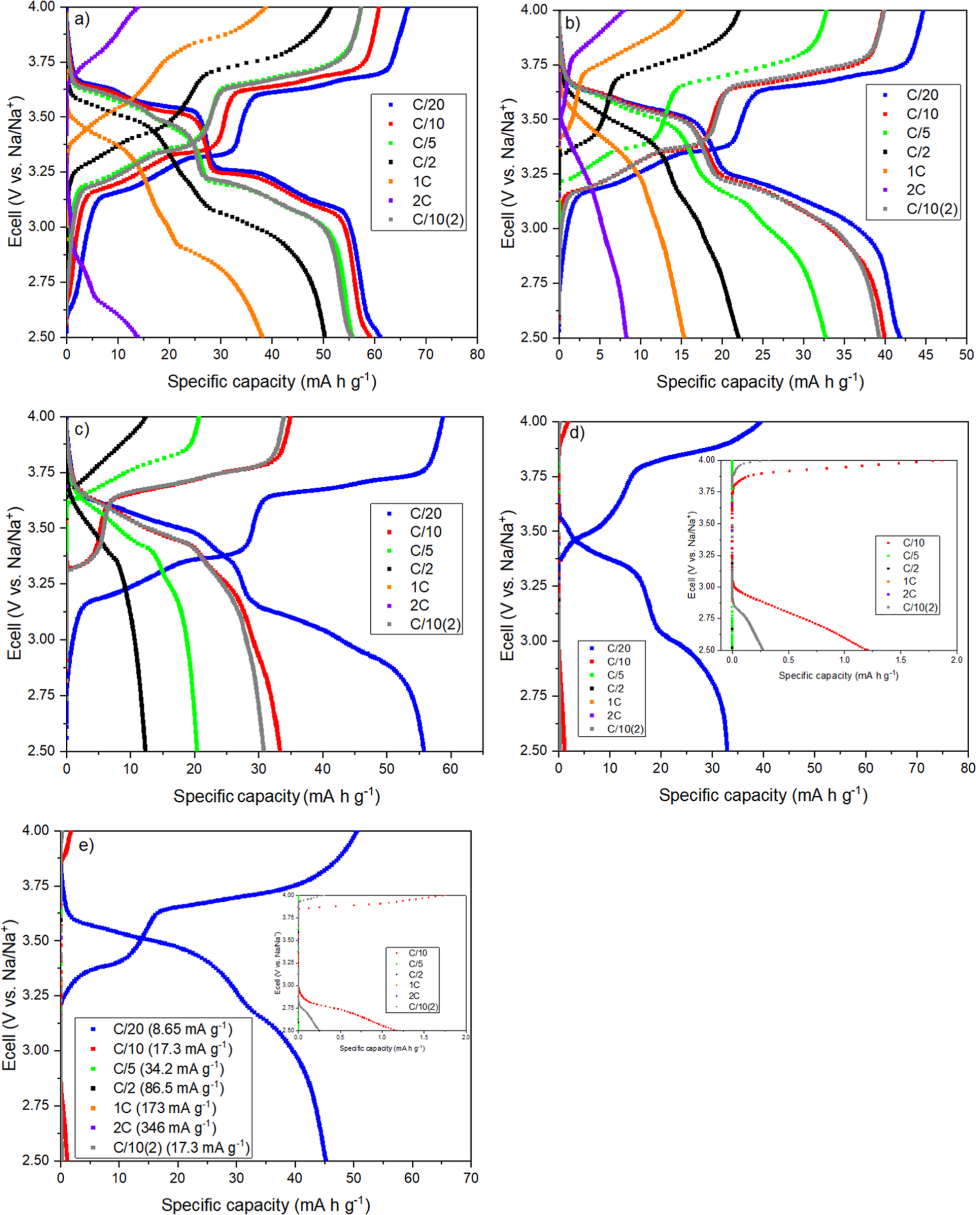
Potential profiles for the last cycle at each C-rate of Na-NNM batteries in the different electrolytes: (a) NaClO_4_ 1.0 mol L^–1^ (EC/PC – 1:1); (b) BMPyr-TFSI 0.5 mol L^–1^; (c) BMPyr-TFSI 1.0 mol L^–1^; (d) BMMI-TFSI 0.5 mol L^–1^; (e) BMMI-TFSI 0.83 mol L^–1^.

Concerning the results with the IL mixtures (Figure 5b–e), the positive electrode exhibited a profile similar to that of the conventional electrolyte in the BMPyr-based ILs (Figure 5b,c) only at low rates (C/20), in contrast to the BMMI-based electrolytes (Figure 5d,e). For the latter, a more drastic change in the potential is observed during cycling at both salt concentrations, possibly associated with kinetic limitations due to the poorer transport properties of the BMMI^+^ cation. This becomes more evident for currents higher than 8.65 mA g^–1^.

The rate capability results shown in Figure 6 can better explain the cycling performance of NNM in the different electrolytes. The results show that NNM achieves its highest specific discharge capacity in the conventional NaClO_4_ electrolyte (blue square), delivering a capacity of 61.2 mA h g^–1^ at C/20 rate. As previously mentioned, NNM has a theoretical capacity of 173 mA h g^–1^ when 2/3 mol of Na^+^ is transferred. However, within the potential range investigated (2.5–4.0 V vs Na/Na^+^), only one-third of the molar amount of Na^+^ is involved in the electrochemical process,^13^ explaining why the capacities are lower than the theoretical value. In this electrolyte, the NNM electrode presented similar performance at C/20, C/10, and C/5 rates, displaying specific discharge capacities of 61.2, 59.1, and 55.7 mA h g^–1^, and a reversible capacity of 55.3 mA h g^–1^ at C/10 rate. This response can be related to the higher ionic conductivity of the electrolyte (7.8 mS cm^–1^)^88^ compared to the ILs, as well as to its lower viscosity (6.0 mPa s).^89^ Additionally, EC and PC present higher dielectric constants than typical organic solvents,^89^ providing a higher degree of dissociation for NaClO_4_ (86.2%).^89^

**Figure 6:**
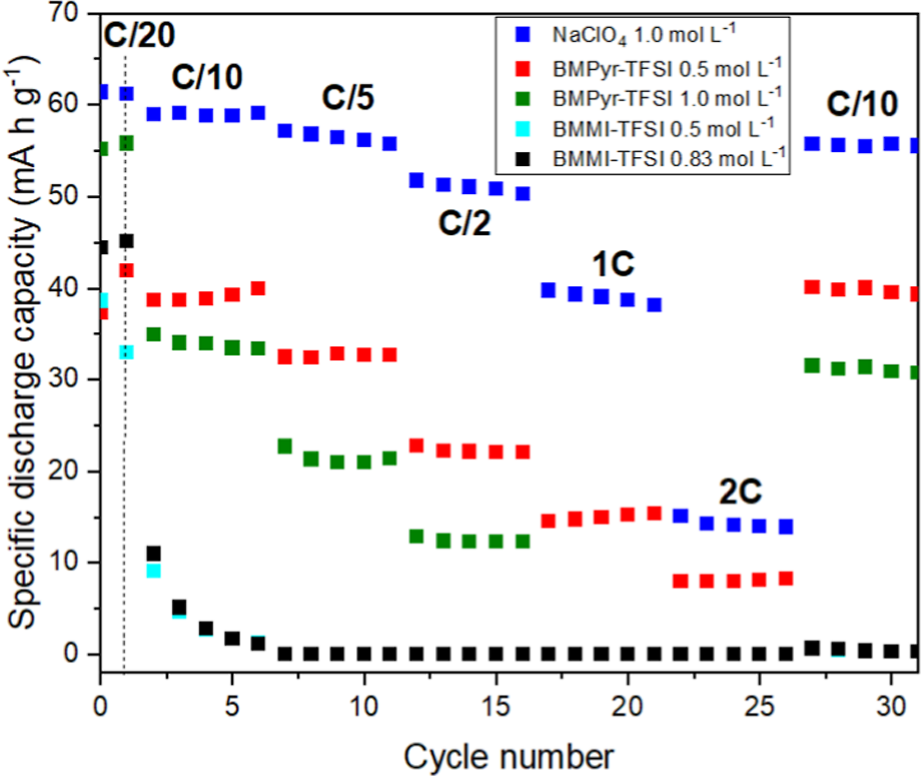
Rate capability of Na-NNM electrodes in the different electrolytes.

Among the ILs, BMPyr-TFSI containing 0.5 mol L^–1^ of NaTFSI (red square) showed the best cyclability and stability, achieving a discharge capacity of 40.0 mA h g^–1^ for the first cycle at C/10 and a capacity of 39.3 mA h g^–1^ for the second cycle at the same rate. BMPyr-TFSI with 1.0 mol L^–1^ of NaTFSI (green square) showed a lower capacity: 33.3 mA h g^–1^ at C/10 rate and a reversible capacity of 30.7 mA h g^–1^. These differences can be ascribed to the better transport properties at lower salt concentrations.

In the case of BMMI-based electrolytes, NNM performed worse at 0.5 (cyan square) and 0.83 mol L^–1^ (black square) of NaTFSI for cycles above C/20 rate. However, comparing the physicochemical properties of the two families of ILs (Figure 2) with the same salt concentration (0.5 mol L^–1^), the values are close and, therefore, a similar performance could have been expected. As discussed in the previous section, however, the BMMI^+^ configuration favors ion–ion interactions that can form neutral aggregates. This reduces the ionicity of IL mixtures and impairs the electrode performance. As the current density increases, the lower ionicity of the imidazolium-based electrolytes appears to significantly impact the cyclability of the electrode at higher C-rates.

The results for the IL-based electrolytes suggest kinetic limitations. To overcome this drawback and further evaluate the feasibility of using BMPyr^+^ and BMMI^+^ as electrolytes, galvanostatic cycles were carried out at very low current densities. For this purpose, the different IL mixtures were subjected to galvanostatic charge–discharge cycles at lower C-rates: C/100, C/50, and C/20. Figure 7 shows the potential profiles for the positive electrode in the various ILs under these conditions. The respective specific currents are presented in the plot legend. The profile for the conventional electrolyte in the last C/20 cycle is also presented (Figure 7e). Table 2 shows the specific discharge capacities obtained from the NNM charge/discharge curves (Figure 7a–d). Additionally, the differential capacity plots (d*Q*/d*E* vs *E*) obtained from the last cycle at the three different rates are presented in Figure 8: C/20 (Figure 8a), C/50 (Figure 8b), and C/100 (Figure 8c), together with the result obtained at C/20 (last cycle) for the conventional organic electrolyte. Although d*Q*/d*E* vs *E* is commonly used to diagnose the state of health of a battery,^90,91^ this type of analysis is also interesting since it provides the fingerprint of the battery system. On the d*Q*/d*E* vs *E* diagrams, each plateau in the potential profile corresponds to a peak, which can be associated with phase changes and intercalation/deintercalation phenomena during the battery cycling.^83,92^

**Figure 7:**
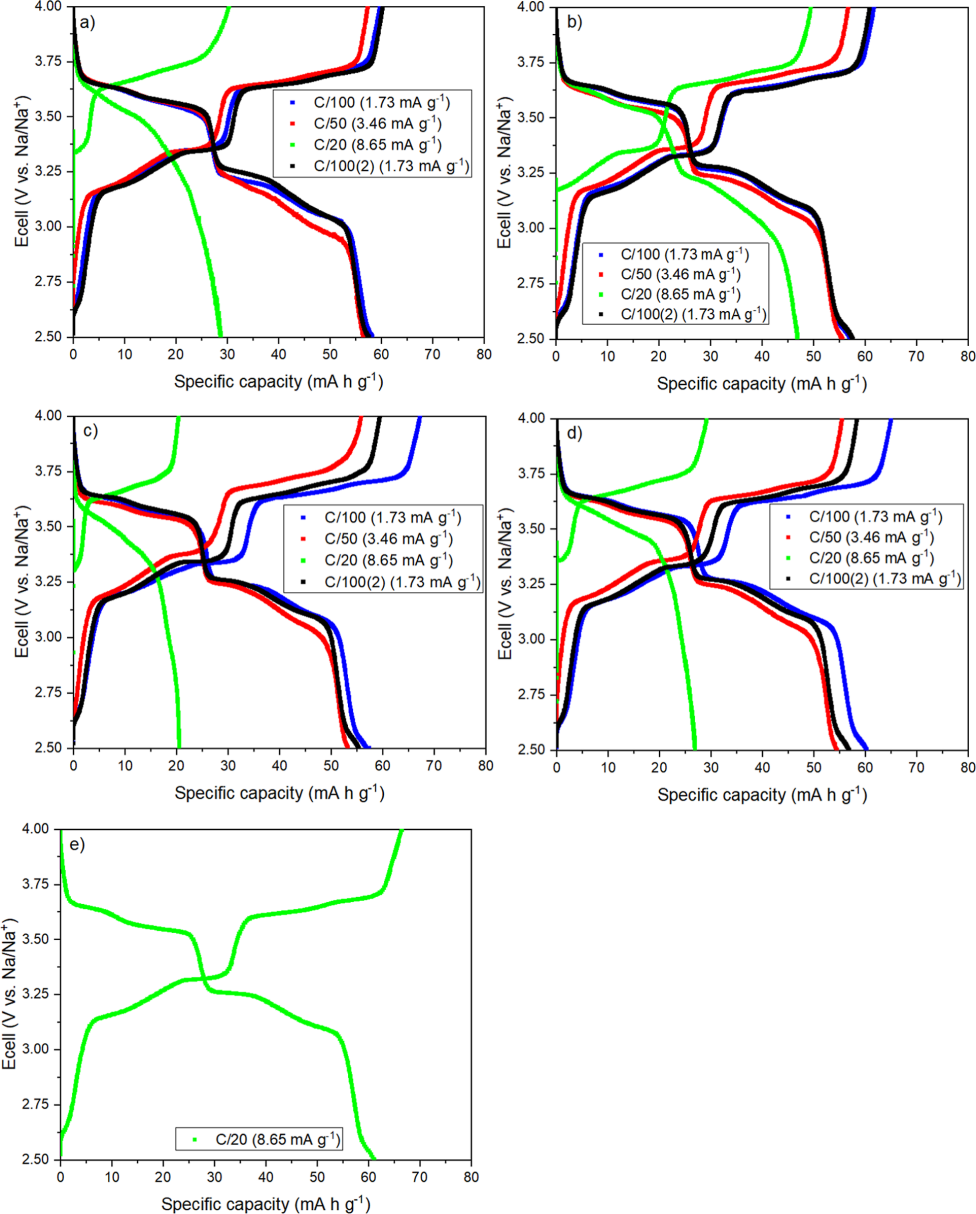
Potential profiles for the last cycle at each C-rate of Na-NNM batteries in the different IL mixtures: (a) BMPyr-TFSI 0.5 mol L^–1^; (b) BMPyr-TFSI 1.0 mol L^–1^; (c) BMMI-TFSI 0.5 mol L^–1^; (d) BMMI-TFSI 0.83 mol L^–1^; (e) NaClO_4_ 1.0 mol L^–1^ (EC/PC—1/1) last cycle at C/20.

**Table 2:** Specific Discharge Capacities Obtained From the Potential Profiles at the C-Rates Presented in Figure 7

		specific discharge capacities (mA h g^–1^)			
cation–anion pair	NaTFSI concentration (mol L^–1^)	C/100	C/50	C/20	C/100
BMPyr-TFSI	0.5	58.24	56.53	28.72	57.78
	1.0	57.21	55.68	47.40	57.61
BMMI-TFSI	0.5	57.49	53.79	20.54	55.38
	0.83	60.33	54.54	26.93	56.83

**Figure 8:**
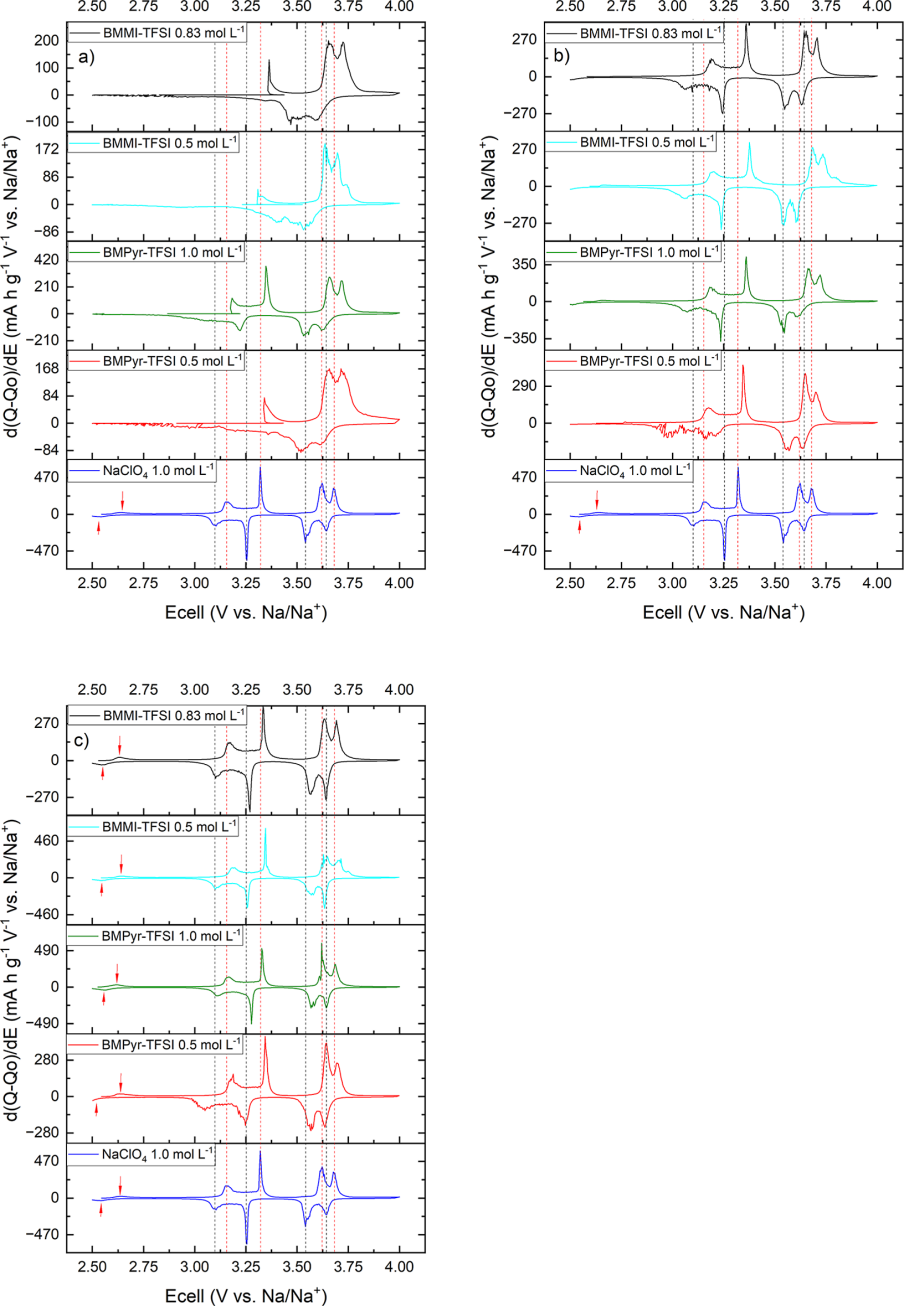
d*Q*/d*E* vs *E* curves for Na-NNM batteries at different rates: (a) C/20; (b) C/50; (c) C/100. In each set, the plot for NaClO_4_ corresponds to the result obtained at the last cycle at C/20. The red dashed lines correspond to the potential shifts for the peaks of NNM in ILs mixture during charge, and the black ones to the potential shifts for the peaks of NNM in ILs mixture during discharge, both compared to the positions in the conventional electrolyte.

The four potential plateaus for NNM in the conventional NaClO_4_ electrolyte (Figure 7e) are reflected by peaks in the d*Q*/d*E* versus *E* plots (blue curve in Figure 8). In addition, a small plateau below 3.0 V vs Na/Na^+^, which is associated with the order rearrangements between Na^+^ and the host vacancies,^82^ is evident as a small shoulder in the d*Q*/d*E* vs *E* plots (indicated by the red arrows). Results for the IL-based electrolytes (Figure 7a–d) show that the cycling rate plays an important role in battery performance. Decreasing the current density resulted inthe electrodes exhibiting a profile similar to that of the conventional electrolyte at C/20. Discharge capacities above 50 mAh g^–1^ were observed at C/100 and C/50 for all conditions (Table 2). Furthermore, the differential capacity plots for both IL families at these rates (Figure 8b,c) show the same four well-defined peaks as in the diagram for the conventional electrolyte. However, compared with perchlorate, potential shifts are observed for the peaks during charge and discharge with increasing C-rate from C/100 to C/50 (positions indicated by the red and black dashed lines, respectively), which can be attributed to increased battery polarization. At C/20 rate, a drastic drop in potential is observed for the ILs during the discharge cycle, resulting in the absence of the characteristic peaks below 3.25 V vs Na/Na^+^ (Figure 8a). This behavior is mainly associated with overpotential due to mass transport limitation.^13^

Similar behavior and close capacity values were observed for all mixtures of BMMI- and BMPyr-based electrolytes at very low rates (C/100 and C/50) (Table 2). These similarities can be attributed to the restricted potential range used in the study, which limits the maximum amount of Na^+^ that can be transferred. Conversely, at C/20 rate, NNM showed a better response in BMPyr-TFSI with 0.5 mol L^–1^ NaTFSI (Figure 7a), compared to BMMI-TFSI with the same salt concentration (Figure 7c), probably due to the better transport properties of the pyrrolidinium cation. The electrode also showed better performance with electrolytes containing higher salt concentrations, i.e., BMPyr-TFSI and BMMI-TFSI with 1.0 mol L^–1^ and 0.83 mol L^–1^ NaTFSI (Figure 7b,d, respectively). This behavior is consistent with the results observed for the rate capability experiments presented in Figure 5, and may be linked to the availability of charge carriers and the sufficiently low reaction rate that allows Na^+^ ions to intercalate.

Figure 9 shows the rate capability (a) and the Coulombic efficiency (CE) (b) versus the number of cycles for NNM in the IL mixtures cycled at low C-rates. Under these conditions, the electrode capacity and cyclability improve for both IL families, resulting in a more stable behavior at C/50 (Figure 9a). Moreover, after five cycles, the CEs were about 98.6 and 98.1% for BMPyr-TFSI with 0.5 and 1.0 mol L^–1^ NaTFSI, and 96.3 and 98.3% for BMMI-TFSI with 0.5 and 0.83 mol L^–1^ NaTFSI (Figure 9b). For the positive electrode, CE is the ratio of discharge to charge capacities, indicating the battery's reversibility, which should be close to 100%. Several factors can reduce the CE, including electrolyte stability, the solid electrolyte interphase properties, the working potential, and the irreversibility of the desodiation/sodiation process, among others.^93,94^

**Figure 9:**
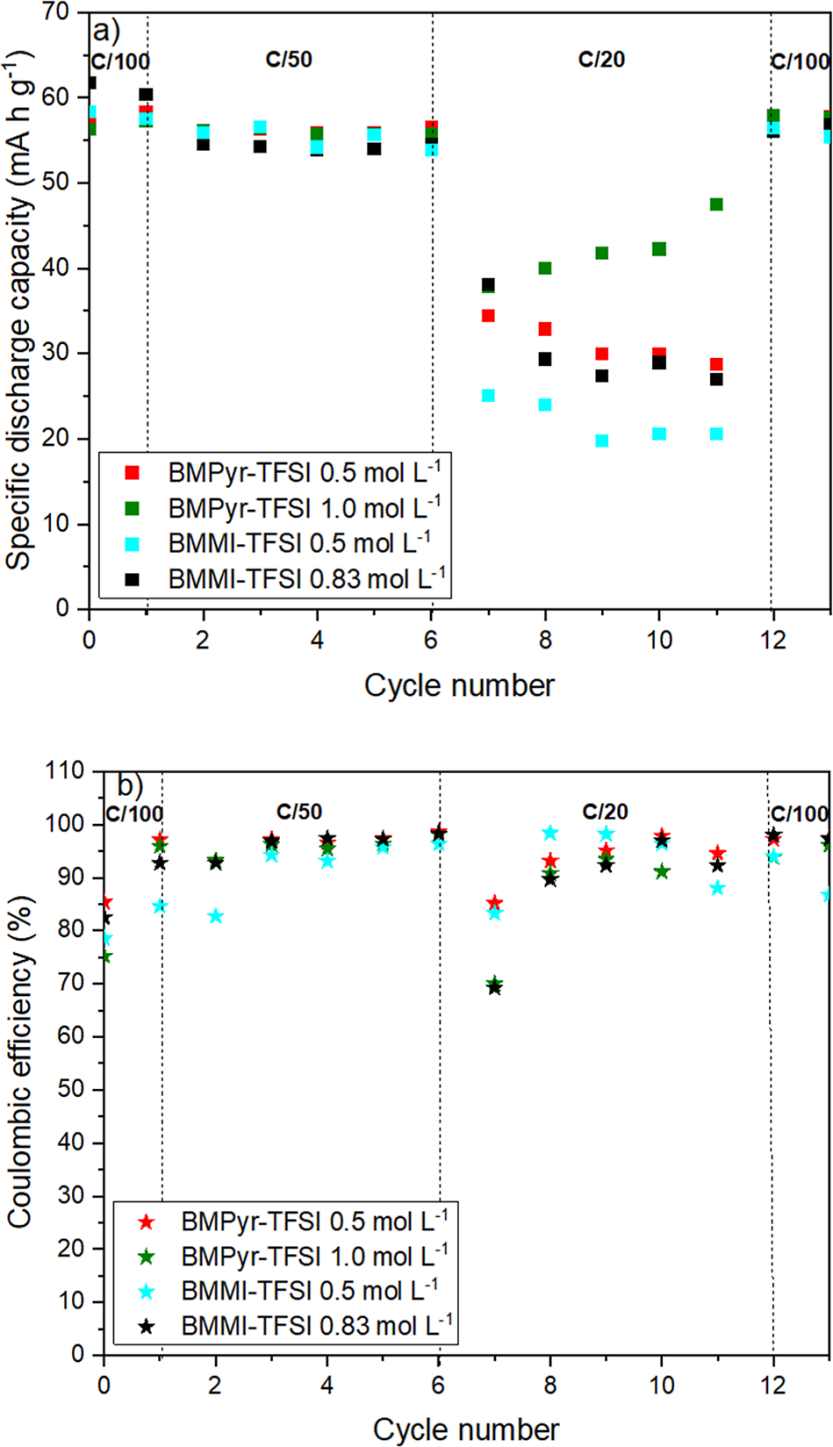
Performance of Na-NNM electrodes in the different electrolytes at low C-rates: (a) rate capability; (b) Coulombic efficiency.

The CEs for NNM in the conventional electrolyte at different C-rates (Figure S5) also show variations over the cycles. After the final cycles at C/10, C/5, C/2, 1C, and 2C, corresponding to the fifth cycle, the CEs were around 97.1, 97.2, 97.7, 98.0, and 99.6%, respectively. These values were compared with those reported by Liu et al.,^37^ whose efficiencies were estimated from potential profiles (range 2.0–4.0 V vs Na/Na^+^) for the active material synthesized at 850 °C. The CEs reported by Liu et al.^37^ were even lower than those found in the present study. After 20 cycles at C/10, C/5, C/2, 1C, and 2C, the efficiencies were estimated around 94.1, 94.1, 94.7, 96.9, and 94.1%, respectively, indicating a certain degree of irreversibility of the synthesized electrode.

For the NNM cycled at C/20 (Figure 9a), the battery capacity drops in all IL mixtures, with the BMMI-TFSI mixtures containing 0.5 and 0.83 mol L^–1^ NaTFSI showing the most pronounced drop. This result is in agreement with those presented in Figure 6, which show that NNM displays a better performance in the BMPyr-based mixtures than in the BMMI-based ones. Nevertheless, a reduction in CEs was observed for all electrolytes with increasing number of cycles for this faster cycling. For instance, after five cycles, NNM reached CEs around 94.5% and 92.2% for BMPyr-TFSI with 0.5 and 1.0 mol L^–1^ NaTFSI, and 88.0% and 92.3% for BMMI-TFSI with 0.5 and 0.83 mol L^–1^ NaTFSI. These decreases may be associated with increased internal resistance and self-consumption.^95,96^

The results discussed in this section demonstrate a clear relationship between the current density of a cycle and the performance of the electrolytes. In fact, kinetic limitations arising from IL transport properties can be overcome by lowering the C-rate. Remarkably, comparable performance of both nitrogen-based ILs to the perchlorate-based electrolyte in terms of Coulombic efficiency and capacity establishes BMPyr-TFSI and BMMI-TFSI ILs as promising electrolytes for NNM. Nevertheless, to understand and evaluate their actual applicability as possible substitutes for the conventional system, electrochemical performance and stability over more extended cycling periods are necessary and will be addressed in future studies.

### Sodium Diffusion Coefficient Determination

Given that only the portion of current carried by sodium ions is relevant in SIBs, and considering that the intercalation process depends fundamentally on this species, it is important that the metal cation demonstrates adequate diffusivity in the electrolyte. Therefore, in addition to the physicochemical properties of ILs, a crucial parameter in the proposal of new electrolytes for sodium-based systems is the diffusion coefficient of Na^+^. As illustrated in Figures S6 and S7, galvanostatic curves and Nyquist diagrams were employed to estimate the sodium diffusion coefficient (*D*_Na_^+^). For the extraction of *D*_Na_^+^ values, the electrochemical response was expressed in terms of the faradaic impedance (*Z*_f_), represented by the sum of the charge transfer resistance (*R*_ct_) and the Warburg impedance (*Z*_w_), as shown in eq 3.^97,98^

3 $$Z_{f}=R_{ct}+Z_{w}$$

*Z*_w_ contribution can be expressed as follows (eq 4):^98^

4 $$Z_{w}=\frac{R\times T}{n^{2}\times F^{2\;}\times \sqrt{j\omega }}\left(\frac{1}{\sqrt{D_{ox}\times C_{ox}\left(0\right)}}+\frac{1}{\sqrt{D_{red\;}{\times C}_{red}\left(0\right)}}\right)$$

In eq 4, *R* is the ideal gases constant (8.314 J mol^–1^ K^–1^), *T* the absolute temperature (K), in this case room temperature (298 K), *n* the number of electrons transferred, *F* the Faraday constant (96,485 C mol^–1^), *D*_ox_ and *D*_red_ correspond to the diffusion coefficients (cm^2^ s^–1^) of the oxidized and reduced species, respectively, while *C*_ox_(0) and *C*_red_(0) stand for the concentration (mol cm^–3^) of the oxidized and reduced species on the electrode surface. Assuming that surface concentrations obey the Nernst law, it is possible to rearrange the Nernst equation and express the ratio *C*_ox_(0)/*C*_red_(0) in terms of eq 5.

5 $$\frac{C_{ox}\left(0\right)}{C_{red}\left(0\right)}=e^{\left(\frac{n\times F}{R\times T}\times \left(E-E^{0}\right)\right)}$$

*E* represents the electrode potential (V) and *E* ^0^ the equilibrium potential (V). It is posited that if the diffusivities of the oxidized and reduced species are equal, the Nernst Equation can be rewritten in terms of the half-wave potential (*E*_1/2_), as shown in eq 6:^99^

6 $$E_{1/2}=E^{0}+\frac{R\times T}{n\times F}\times \ln\left(\sqrt{\frac{D_{red}}{D_{ox}}}\right)$$

By replacing eqs 5 and 6 in eq 4, the equation for the diffusion impedance can be rewritten (eq 7)­

7 $$Z_{w}=\frac{4\times R\times T}{n^{2}\;{\times F}^{2}\;{\times C}_{ox}\times \;\sqrt{j\omega \times D_{ox}}}\cosh^{2}\left(\frac{n\times F\times \left(E-E_{1/2}\right)}{2\times R\times T}\right)$$

For a finite length linear diffusion, the boundary conditions for solving the second Fick’s law are adapted, thus eq 7 turns to eq 8

8 $$Z_{w}=\frac{R\times T}{n^{2}{\times F}^{2}\times \sqrt{j\omega }}\left(\frac{\coth\left(\delta_{N}\times \sqrt{\frac{j\omega }{D_{ox}}}\right)}{\sqrt{D_{ox}{\times C}_{ox}\left(0\right)}}+\frac{\coth\left(\delta_{N}\times \sqrt{\frac{j\omega }{D_{red}}}\right)}{\sqrt{D_{red}\;{\times C}_{red}\left(0\right)}}\right)$$

δ_*N*_ represents the diffusion layer thickness. As the diffusivities of the oxidized and reduced species are equals $({D_{ox}=D}_{red}=D$ ), eq 8 can be reduced to eq 9

9 $$Z_{w}=\frac{4\times R\times T}{n^{2}{\times F}^{2}\;{\times C}_{ox}\times \sqrt{D}}\cosh^{2}\left(\frac{n\times F\times \left(E-{\;E}_{1/2}\right)}{2\times R\times T}\right)\left(\frac{\coth\left(\delta_{N}\times \sqrt{\frac{j\omega }{D}}\right)}{\sqrt{j}\omega }\right)$$

For describing a nonideal behavior of the diffusion impedance, a parameter similar to a CPE that accounts for such response, represented by the symbol (Φ_w_), can be introduced in eq 9,^100,101^ resulting in eq 10

10 $$Z_{w}=\frac{4\times R\times T}{n^{2}{\times F}^{2}\;{\times C}_{ox}\times \sqrt{D}}\cosh^{2}\left(\frac{n\times F\times \left(E-E_{1/2}\right)}{2\times R\times T}\right)\frac{\delta_{N}}{{\sqrt{D}\times \left(\delta_{N}\times \sqrt{\frac{j\omega }{D}}\right)}^{\Phi_{w}}}\coth{\left(\delta_{N}\times \sqrt{\frac{j\omega }{D}}\right)}^{\Phi_{w}}$$

To fit the experimental data, the Measurement Model software was used.^102^ The fitting results, as well as the parameters, are presented in the SI section (Figure S8 and Table S3). Table 3 shows the *D*_Na_^+^ estimations for the different electrolytes. For the perchlorate-based electrolyte, *D*_Na_^+^ was estimated at 1.3 × 10^–7^ cm^2^ s^–1^. Since most studies on metallic ion diffusion in electrolytes for metal-ion systems are usually based on the use of pulsed field gradient (PEG)-NMR measurements,^103–106^ resulting in values associated with the self-diffusion of the species,^103^ a direct comparison between the values presented in Table 3 with those reported in the literature becomes quite challenging. However, through molecular dynamics simulations for 1.0 mol L^–1^ NaClO_4_ in EC electrolyte, Friedman^107^ estimated a Na^+^ diffusion coefficient of 2.9 × 10^–7^ cm^2^ s^–1^, a value close to that found in the present work.

**Table 3:** Sodium Diffusion Coefficients Calculated from eq 3

electrolyte	salt concentration (mol L^–1^)	sodium diffusion coefficient (cm^2^ s^–1^)
NaClO_4_ in EC/PC (1:1 v/v)	1.0	1.30 × 10^–7^
BMPyr-TFSI	0.5	1.41 × 10^–7^
	1.0	5.55 × 10^–8^
BMMI-TFSI	0.5	2.70 × 10^–7^
	0.83	1.83 × 10^–8^

Concerning the ILs mixtures, the increase of NaTFSI concentration reduced the diffusivity of Na^+^. For BMPyr-TFSI with 0.5 mol L^–1^ of NaTFSI, the estimated sodium diffusion coefficient was 1.41 × 10^–7^ cm^2^ s^–1^, while for 1.0 mol L^–1^ the value was almost 2.5 times lower (5.55 × 10^–8^ cm^2^ s^–1^). In the case of imidazolium-based electrolytes, the estimated diffusion coefficient for BMMI-TFSI with 0.5 and 0.83 mol L^–1^ of NaTFSI were 2.7 × 10^–7^ and 1.83 × 10^–8^ cm^2^ s^–1^, respectively. These observations are in agreement with previous studies that reported analogous behaviors in the presence of varying concentrations of metallic salts in ILs.^68,71,108,109^ These studies attributed this behavior to alterations in solvation dynamics, due to the elevated NaTFSI content.^71^ Although higher salt concentrations have been shown to increase the metallic cation transfer number,^110^ defined as the proportion of the total current carried by the metallic ion, this increase in the density of ionic species leads to an increase in Na-TFSI interactions, as previously discussed in the section on physicochemical properties of ILs. The increase in Na-TFSI interactions explains the inhibition of sodium mobility at higher NaTFSI concentrations. A comparison of the diffusion coefficient values estimated in the ILs containing BMPyr^+^ and BMMI^+^ at the same salt concentration (0.5 mol L^–1^ of NaTFSI), suggests that a larger difference between the *D*_Na_^+^ values would be expected, especially considering the overall superior cycling performance shown by NNM in the BMPyr-TFSI IL at higher C-rates (Figures 5 and 6). However, the efficiency of an electrolyte depends not only on the diffusivity of charge carriers through the medium, but also on the density of carriers available for diffusion. As illustrated by the Walden’s plot (Figure 3), for a concentration of 0.5 mol L^–1^ of NaTFSI, the density of aggregates formed in the imidazolium-based IL is greater than that observed in the pyrrolidinium-based electrolyte. Thus, despite the physicochemical properties of the two mixtures being analogous, the enhanced tendency to form aggregates in the presence of BMMI^+^ seems to play a significant role, influencing the performance of the electrolyte.

### CEI Properties

In addition to considerations of battery stability and cyclability, the solid electrolyte interphase formed between the cathode and the electrolyte plays a crucial role. This interphase obstructs the electronic transport of the active material to the electrolyte while allowing the passage of metal cations during intercalation and deintercalation.^111^ Moreover, it modulates the reaction rate in the battery.^112^ One way to access information about the properties of the CEI is through the EIS technique, in which it is possible to discriminate the contribution of this passive film in relation to other systems for the electrochemical response of the battery.

Figure 10 shows the Nyquist plots obtained prior to and following cycling in the different electrolytes. After cycling, the spectra were obtained at the discharged state of the NNM electrode, i.e., at 2.5 V vs Na/Na^+^. For the conventional electrolyte (Figure 10a), the diagram prior to cycling can be decrypted into two regions. In the high-to-medium frequency range (10 kHz–10 Hz), the semicircle is indicative of Na^+^ transfer across the interface between the active material surface and the electrolyte,^113^ mainly associated with charge-transfer resistance.^114,115^ At lower frequencies (below 2 Hz), the response of the system becomes more resistive, which could indicate limitations in mass transfer. After the two cycles at C/20 (illustrated by the filled blue curve), an improvement in the electrode response was observed, accompanied by a decrease in impedance, possibly related to the formation of a stable CEI. Previous works^89,116^ on the use of different organic solvents combinations for Na-ion batteries have also shown that the combination of EC/PC as a solvent for NaClO_4_ enhances battery performance, attributed to the formation of a solid interphase with superior properties.

**Figure 10:**
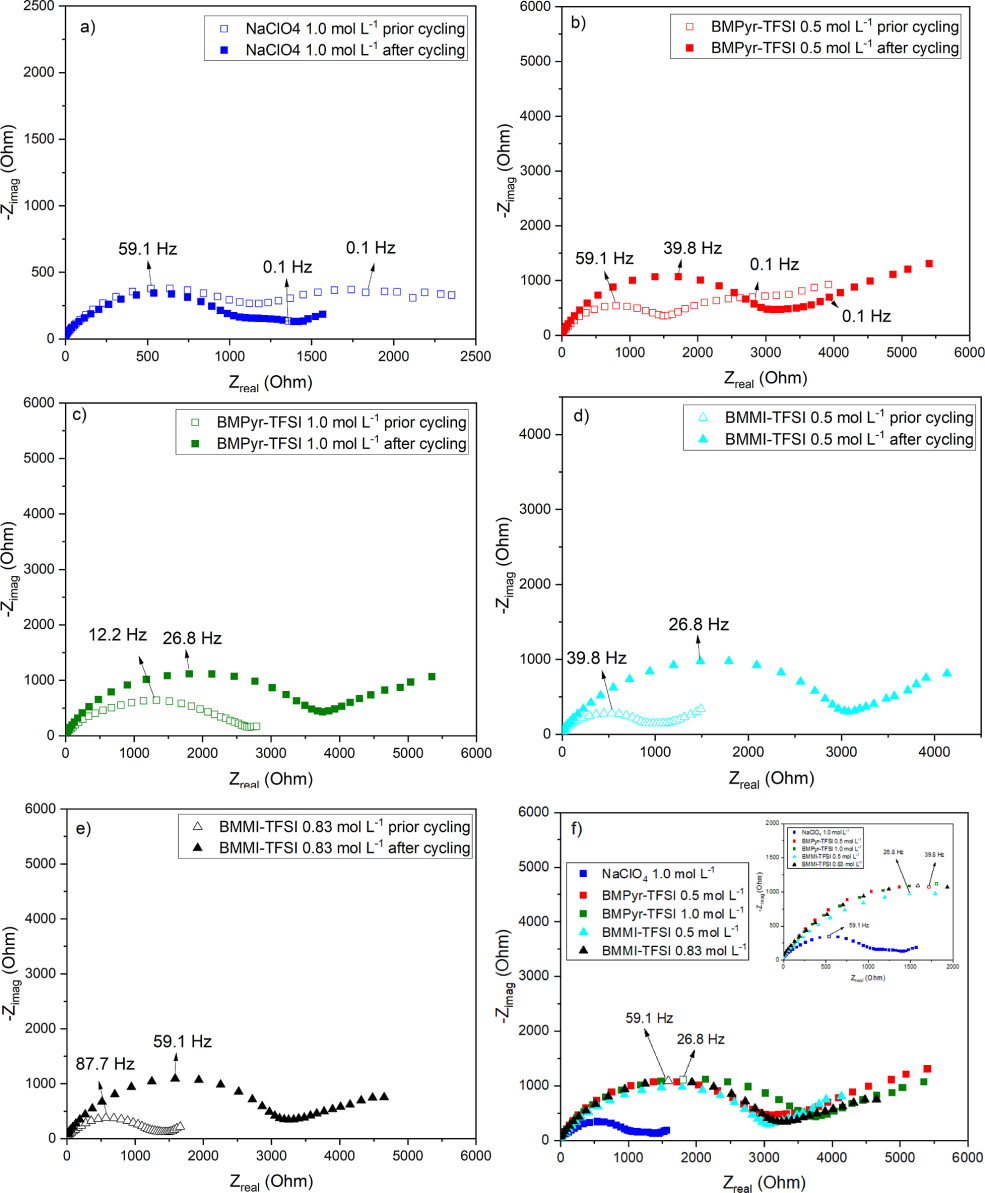
Nyquist plots obtained in the different electrolytes prior to and after cycling: (a) NaClO_4_ 1.0 mol L^–1^; (b) BMPyr-TFSI 0.5 mol L^–1^; (c) BMPyr-TFSI 1.0 mol L^–1^; (d) BMMI-TFSI 0.5 mol L^–1^; (e) BMMI-TFSI 0.83 mol L^–1^; (f) comparison between the different electrolytes after cycling.

In contrast with the perchlorate-based electrolyte, the NNM tested in the IL mixtures (Figure 10b–e) exhibited an overall increase in impedance after the two cycles at C/50. This response will be further discussed in the following paragraph. The analysis of the spectra showed a semicircle from high to medium frequencies, followed by a depressed arc at the low-frequency domain (below 1 Hz). This latter finding indicates a deviation from the ideal Warburg impedance, which is likely attributable to a diffusion-controlled process in bounded or irregular geometries.^117,118^ Moreover, increasing the salt concentration of the ILs slightly increased the cell impedance, likely attributable to the electrolytes’ poorer transport properties.

Figure 10f provides a comparison of the Nyquist plots for NNM after cycling in the various electrolytes. The positive electrode demonstrated the lowest impedance in perchlorate-based electrolyte (blue square curve), in contrast to all batteries tested in the ILs mixtures, which exhibited higher impedances. Do et al.^119^ also performed EIS experiments to gather information on the properties of the P2–Na_0.6_Co_0.1_Mn_0.9_O_2+*z*_ positive electrode, as well as the formed CEI, both in the 1.0 mol L^–1^ NaClO_4_ in EC/PC and in the 0.5 mol L^–1^ NaTFSI in BMPyr-TFSI electrolytes. The EIS spectra were recorded after the first, 30th, and 500th galvanostatic charge–discharge cycle. While the response was better in NaClO_4_ after 30 cycles, a decrease in impedance was observed between the first and the 30th cycle for the electrode tested in the IL. The authors suggested that this behavior may result from insufficient electrode wetting by the electrolyte or the formation of a more conductive CEI during the initial cycles. However, it is important to note that, in the present study, cycling was carried out at very low specific currents (3.46 mA g^–1^ compared to 50 mA g^–1^ in Do et al.), which would favor better electrode wetting by the electrolytes. Therefore, the observed impedance increase in all mixtures could be indicative of the formation of a CEI with more resistive characteristics than that formed in the conventional electrolyte. The formation of this passive layer is primarily attributed to the decomposition of IL anion,^119,120^ suggesting that the chemical composition, as well as the final properties, will be similar for the different mixtures, thus explaining the comparable responses observed in Figure 10f for the ILs.

## Conclusions

The electrochemical performance of two families of nitrogen-based ILs as electrolytes for the Na_0.67_Ni_0.33_Mn_0.67_O_2_ (NNM) positive electrode for sodium–ion batteries (SIBs) was evaluated. To supplement the existing body of knowledge, the physicochemical (density, viscosity, and conductivity) and thermal properties of the neat electrolytes and their mixtures with different concentrations of NaTFSI were determined and compared. Among the neat ILs, BMPyr-TFSI exhibited superior physicochemical properties, with higher conductivity and lower viscosity compared to BMMI-TFSI. Furthermore, the addition of salt was detrimental to the electrolyte properties, resulting in increased density and viscosity while concurrently reducing the ionic conductivity.

Concerning the electrochemical behavior, galvanostatic cycling results demonstrated that, when cycled at high rates (above C/20), the NNM positive electrode showed significantly worse behavior in the ILs when compared with the conventional electrolyte (NaClO_4_). Moreover, the EIS experiments indicated the potential formation of a CEI with enhanced resistive characteristics in the presence of the IL mixtures, which could impede the intercalation/deintercalation process of the metal cation. A reduction in current density (C/50 and C/100) has been shown to enhance the performance of the IL-based electrolytes, apparently suppressing the kinetic limitations due to poorer transport properties. At these low rates, both pyrrolidinium and imidazolium-based ILs showed capacities and efficiencies comparable to those of the perchlorate-based electrolyte, suggesting they may be suitable alternatives as safe electrolytes.

## Supplementary Material



## Notes

The X-ray diffraction pattern for the pristine NNM particles is presented in Figure S1. Figures S2 and S3 present the SEM image of the electrode prior to the experiments (backscattered electrons) and the EDS spectrum of the NNM particles, respectively. Figure S4 shows the linear representation of the Walden plot for the different mixtures of nitrogen-based ILs. Table S1 presents the parameters obtained from the viscosity curves fitting with VFT eq (1), and Table S2 shows the parameters obtained from the conductivity curves fitting with the VFT eq (2). Figure S5 presents the Coulombic efficiency of NNM in 1.0 mol L^–1^ NaClO_4_ (EC/PC) at different C-rates. Figure S6 presents the galvanostatic cycles of sodium deposition on copper electrodes prior to the acquisition of EIS spectra. Figure S7 shows the impedance spectra after galvanostatic treatment. The fitting results for the extraction of sodium diffusion coefficients, in the different electrolytes, from experimental impedance data are presented in Figure S8. Table S3 shows the parameters obtained for the fitting of the EIS diagrams (PDF)
