# Supplementary material for: Pyrrolidinium and Imidazolium-Based Ionic Liquids as Electrolytes for Na0.67Ni0.33Mn0.67O2 Positive Electrode for Sodium–Ion Batteries
Source: J Phys Chem B. 2026 Jan 28;130(6):1915–32. doi: 10.1021/acs.jpcb.5c07871 (PMC12908124; doi:10.1021/acs.jpcb.5c07871)
Supplement: Supplementary file 1 [file jp5c07871_si_001.pdf]

Pyrrolidinium and Imidazolium-Based Ionic Liquids as electrolytes for $Na_{0.67}Ni_{0.33}Mn_{0.67}O_2$ positive electrode for Sodium-Ion batteries

Leandro S. Domingues,[1,2] Roberto M. Torresi,[3] Vincent Vivier,[2] Mireille Turmine*[,2],

Vitor L. Martins*[,3], Hercilio G. de Melo*[,1]

[1] Departamento de Engenharia Metalúrgica e de Materiais, Universidade de São Paulo, Av. Professor Mello Moraes, 2463 - Butantã, São Paulo - SP, 05508-030, Brazil

[2] Sorbonne Université, CNRS, Laboratoire de Réactivité de Surface (LRS), 4 Place Jussieu, 75005, Paris, France

[3] Departamento de Química Fundamental, Instituto de Química, Universidade de São Paulo, Av. Prof. Lineu Prestes 748, - Butantã, São Paulo – SP, 05508-000, Brazil

Supporting Information

Active material and electrode characterization

Figures S1-S3 show the structural and morphological analyses of the pristine NNM material. Figure S1 presents the XRD pattern obtained for the powder material after synthesis, and Figures S2 and S3 show the SEM micrography of the electrode before electrochemical tests and the EDS analysis of the material.

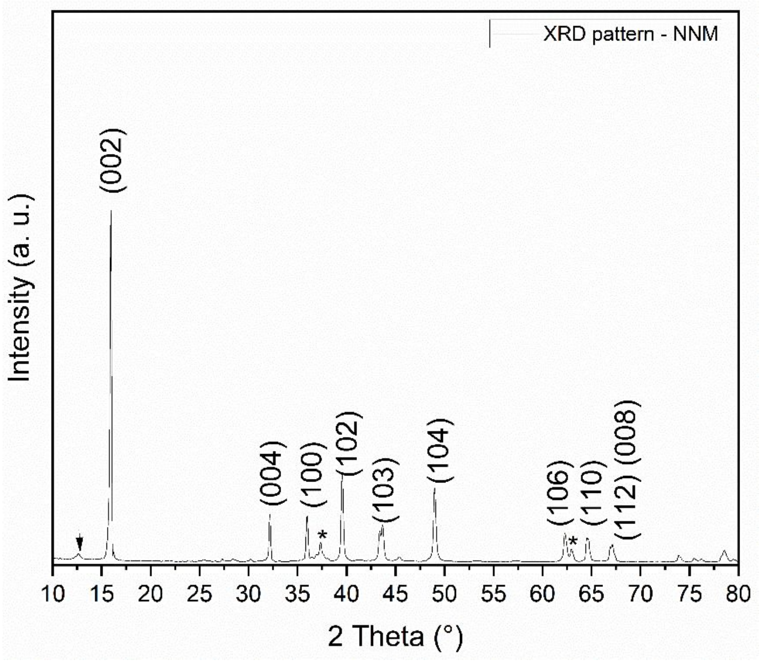

Figure S1. *X-Ray diffraction pattern for the pristine NNM particles.*

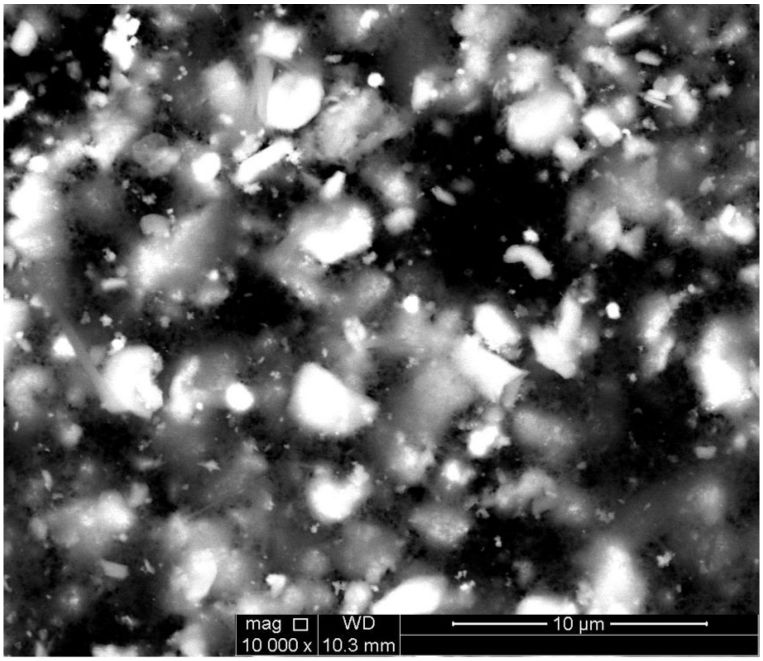

Figure S2. SEM image of the electrode before the experiments (backscattered electrons).

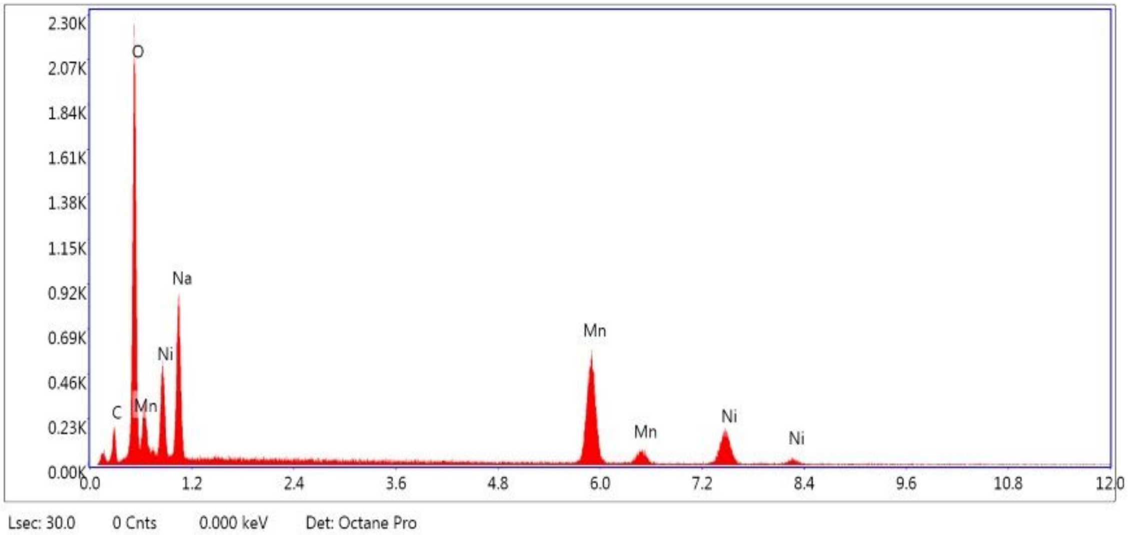

Figure S3. EDS spectrum of the NNM particles.

The crystallographic plans shown in Figure S1 are those typically found in the P2-type prismatic structure reported in the literature[13,80,83,121,122]. In this layered system, the transition metal oxides form octahedral arrangements in which Na^+ occupies the prismatic sites. Contrary to the diffraction pattern of sintered NNM obtained at 850 ˚C by Liu et al.[37] two peaks were observed near 37.5˚ and 62.5˚ (represented by the asterisks), associated with NiO impurities on the oxide layer compound[18,37]. Moreover, a small peak can be observed near 12.5˚ (black arrow), associated with a small remnant of hydrated phase[18]. SEM micrograph reveals a distribution of fine NNM particles with sizes of a few micrometers (Figure S2), as confirmed by EDS analysis (Figure S3).

Physicochemical properties

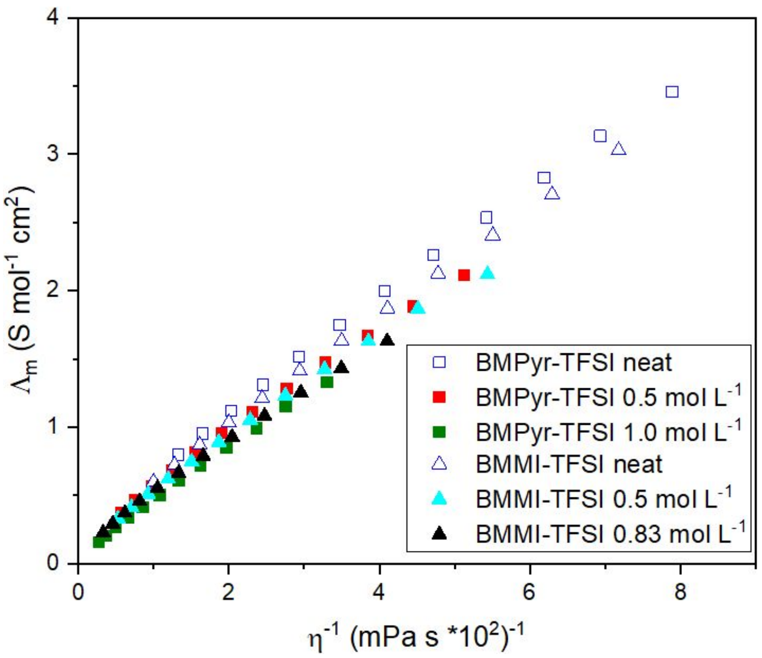

Figure S4. *Linear representation of the Walden plot for the different mixtures of nitrogen-based ILs.*

Table S1. Parameters obtained from the viscosity curves fitting with the VFT Equation (1).

Cation-Anion pair	NaTFSI concentration (mol L^{-1})	η_0 (mPa s)	T_0 (K)	D_{VFT}	R^2
BMPyr-TFSI	--	0.16±0.008	163.05±1.20	5.1±0.1	0.99999
	0.5	0.15±0.006	176.61±0.74	4.83±0.08	0.99999
	1.0	0.12±0.021	174.79±2.99	5.6±0.4	0.99996
BMMI-TFSI	--	0.17±0.009	174.81±1.19	4.5±0.1	0.99999
	0.5	0.14±0.013	179.85±1.76	4.8±0.2	0.99998
	0.83	0.15±0.004	186.07±0.44	4.59±0.05	0.99999

Table S2. Parameters obtained from the conductivity curves fitting with the VFT Equation (2).

Cation-Anion pair	NaTFSI concentration (mol L^{-1})	σ_0 (S cm^{-1})	T_0 (K)	D_{VFT}	R^2
	--	0.38±0.033	162.26±3.61	4.2±0.3	0.99997

**BMPyr-TFSI**	0.5	0.36±0.016	171.37±1.64	4.1±0.1	0.99999
	1.0	0.58±0.058	171.66±3.10	5.1±0.3	0.99998
**BMMI-TFSI**	--	0.78±0.030	145.25±1.40	6.3±0.2	0.99999
	0.5	0.74±0.072	158.18±3.37	5.7±0.3	0.99998
	0.83	0.49±0.047	170.15±3.18	4.8±0.3	0.99998

Electrochemical characterization

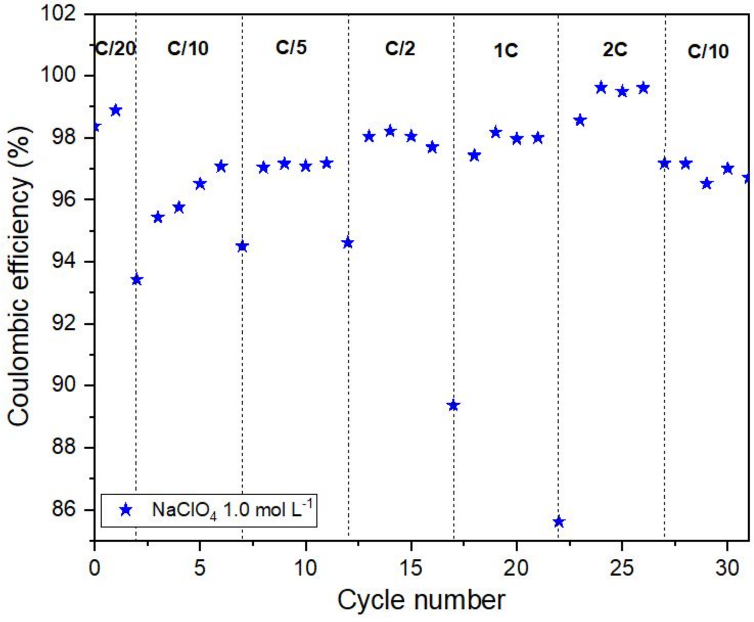

Figure S5. *Coulombic efficiency of NNM in 1.0 mol L^{-1} $NaClO_4$ (EC:PC) at different C-rates.*

Sodium diffusion coefficient determination

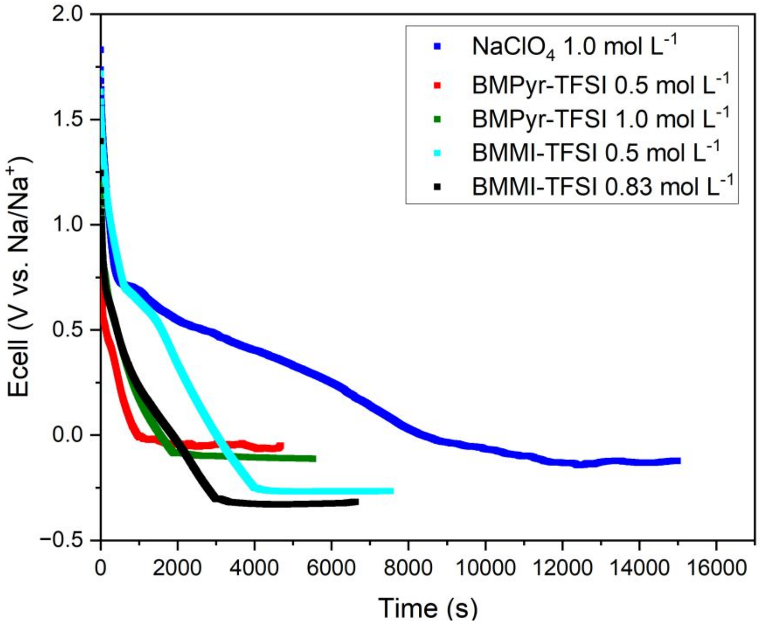

Figure S6. *Galvanostatic cycles of sodium deposition on copper electrodes.*

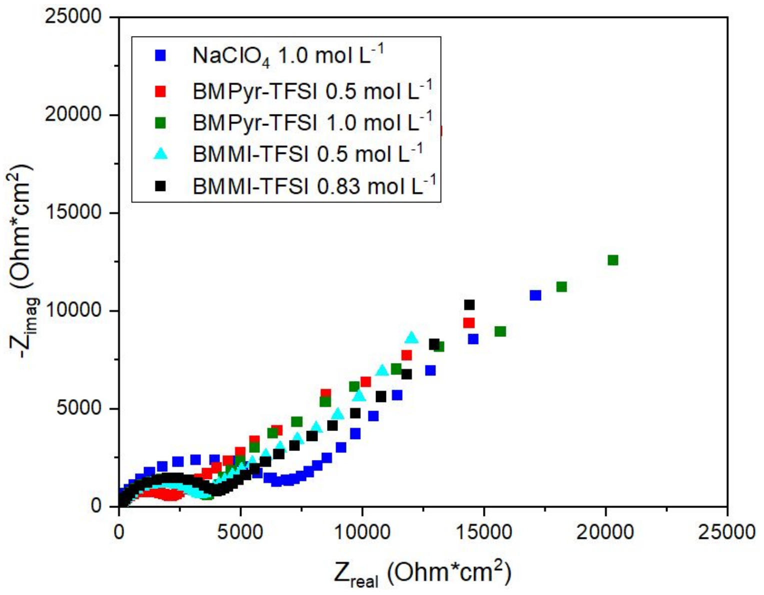

Figure S7. *Impedance spectra obtained after galvanostatic treatment.*

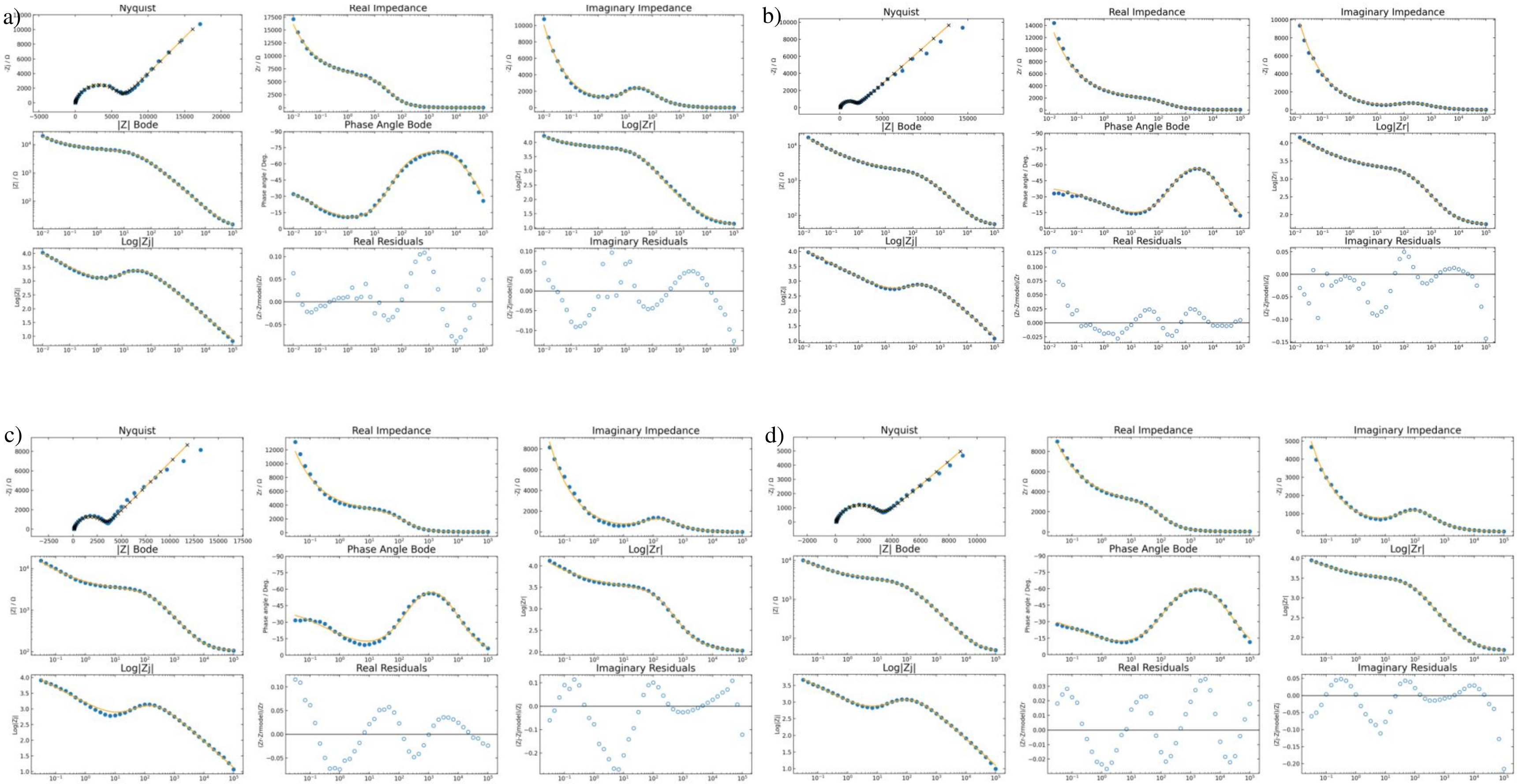
a)
Nyquist
Real Impedance
Imaginary Impedance
|Z| Bode
Phase Angle Bode
Log|Zr|
Log|Zj|
Real Residuals
Imaginary Residuals
b)
Nyquist
Real Impedance
Imaginary Impedance
|Z| Bode
Phase Angle Bode
Log|Zr|
Log|Zj|
Real Residuals
Imaginary Residuals
c)
Nyquist
Real Impedance
Imaginary Impedance
|Z| Bode
Phase Angle Bode
Log|Zr|
Log|Zj|
Real Residuals
Imaginary Residuals
d)
Nyquist
Real Impedance
Imaginary Impedance
|Z| Bode
Phase Angle Bode
Log|Zr|
Log|Zj|
Real Residuals
Imaginary Residuals

e)

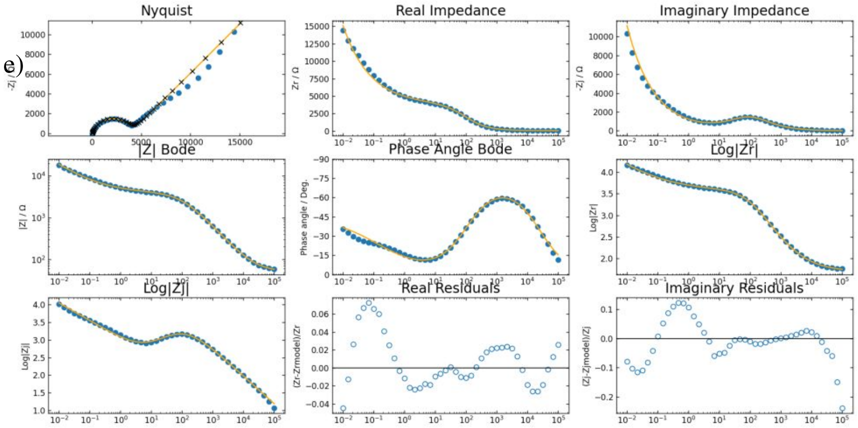

Figure S8. *Fitting results for the different EIS diagrams using Eqs. 3 and 10: a) $NaClO_4$ 1.0 mol L^{-1}; b) BMPyr-TFSI 0.5 mol L^{-1}; c) BMPyr-TFSI 1.0 mol L^{-1}; d) BMMI-TFSI 0.5 mol L^{-1}; e) BMMI-TFSI 0.83 mol L^{-1}.*

Table S3. Parameters obtained from the fitting performed in the EIS diagrams to estimate the diffusion coefficient (fitting errors in parenthesis).

Electrolyte	Salt concentration (mol L^{-1})	Sodium diffusion coefficient (cm^2 s^{-1})	Delta	Eta	Phi	Electrolyte resistance Ohm cm^2)	Double-layer capacitance (F cm^{-2} $S^{(n-1)}$	Alfa	Charge Transfer resistance (Ohm cm^2)
$NaClO_4$ in EC:PC (1:1 v/v)	1.0	$1.30*10^{-7}$ ($1.94*10^{-8}$)	0.03 (0)	0.1(0)	1.0 (0.01)	11.14 (0.26)	$1.72*10^{-6}$ ($4.60*10^{-8}$)	0.84 (0.003)	6100.6 (84.96)
BMPyr-TFSI	0.5	$1.41*10^{-7}$ ($9.49*10^{-9}$)	0.03 (0)	0.1(0)	0.9 (0.01)	50.21 (0.67)	$1.25*10^{-6}$ ($5.57*10^{-8}$)	0.82 (0.005)	1824.81 (27.15)
	1.0	$5.55*10^{-8}$ ($1.22*10^{-8}$)	0.03 (0)	0.1(0)	1.0 (0.01)	105.96 (1.92)	$8.70*10^{-7}$ ($6.54*10^{-8}$)	0.85 (0.008)	3077.5 (72.82)
BMMI-TFSI	0.5	$2.70*10^{-7}$ ($2.46*10^{-8}$)	0.03 (0)	0.1(0)	0.9 (0.01)	43.46 (0.47)	$1.61*10^{-6}$ ($4.61*10^{-8}$)	0.81 (0.003)	3033.74 (36.93)

	0.83	$1.83*10^{-8}$ ($0.67*10^{-8}$)	0.03 (0)	0.1(0)	1.0 (0.01)	134.60 (2.71)	$0.91*10^{-6}$ ($1.12*10^{-8}$)	0.85 (0.003)	3928.4 (38.3)

REFERENCES

(13) Mao, J.; Liu, X.; Liu, J.; Jiang, H.; Zhang, T.; Shao, G.; Ai, G.; Mao, W.; Feng, Y.; Yang, W.; Liu, G.; Dai, K. P2-Type Na 2/3 Ni 1/3 Mn 2/3 O 2 Cathode Material with Excellent Rate and Cycling Performance for Sodium-Ion Batteries . J Electrochem Soc 2019, 166 (16), A3980–A3986. https://doi.org/10.1149/2.0211916jes.

(18) Doubaji, S.; Ma, L.; Asfaw, H. D.; Izanzar, I.; Xu, R.; Alami, J.; Lu, J.; Wu, T.; Amine, K.; Edström, K.; Saadoune, I. On the P2-NaxCo1-y(Mn2/3Ni1/3)YO2 Cathode Materials for Sodium-Ion Batteries: Synthesis, Electrochemical Performance, and Redox Processes Occurring during the Electrochemical Cycling. ACS Appl Mater Interfaces 2018, 10 (1), 488–501. https://doi.org/10.1021/acsami.7b13472.

(37) Liu, G.; Wen, L.; Li, Y.; Kou, Y. Synthesis and Electrochemical Properties of P2-Na2/3Ni1/3Mn2/3O2. Ionics (Kiel) 2015, 21 (4), 1011–1016. https://doi.org/10.1007/s11581-014-1249-2.

(80) Wang, K.; Yan, P.; Sui, M. Phase Transition Induced Cracking Plaguing Layered Cathode for Sodium-Ion Battery. Nano Energy 2018, 54 (September), 148–155. https://doi.org/10.1016/j.nanoen.2018.09.073.

(83) Lu, Z.; Dahn, J. R. In Situ X-Ray Diffraction Study of P2-Na[Sub 2/3][Ni[Sub 1/3]Mn[Sub 2/3]]O[Sub 2]. J Electrochem Soc 2001, 148 (11), A1225. https://doi.org/10.1149/1.1407247.

(121) Zhang, Y. Y.; Zhang, S. J.; Li, J. T.; Wang, K.; Zhang, Y. C.; Liu, Q.; Xie, R. S.; Pei, Y. R.; Huang, L.; Sun, S. G. Improvement of Electrochemical Properties of P2-Type Na 2/3 Mn 2/3

Ni 1/3 O 2 Sodium Ion Battery Cathode Material by Water-Soluble Binders. Electrochim Acta 2019, 298, 496–504. https://doi.org/10.1016/j.electacta.2018.12.089.

(122) Hu, H.; Tang, K.; Cao, S.; Yang, X.; Wang, X. Synthesis and Electrochemical Properties of P2–Na2/3[Ni1/3Mn2/3]O2 Microspheres as Cathode Materials for Sodium-Ion Batteries. J Alloys Compd 2021, 859. https://doi.org/10.1016/j.jallcom.2020.157768
